# A four-electron Zn-I_2_ aqueous battery enabled by reversible I^−^/I_2_/I^+^ conversion

**DOI:** 10.1038/s41467-020-20331-9

**Published:** 2021-01-08

**Authors:** Yiping Zou, Tingting Liu, Qijun Du, Yingying Li, Haibo Yi, Xing Zhou, Zhuxin Li, Lujie Gao, Lan Zhang, Xiao Liang

**Affiliations:** 1 State Key Laboratory of Chem/Bio-Sensing and Chemometrics, College of Chemistry and Chemical Engineering, Hunan University, Changsha, 410082 P.R. China; 2 Advanced Catalytic Engineering Research Center of the Ministry of Education, Hunan University, Changsha, 410082 P.R. China

**Keywords:** Batteries, Batteries, Batteries

## Abstract

Electrochemically reversible redox couples that embrace more electron transfer at a higher potential are the eternal target for energy storage batteries. Here, we report a four-electron aqueous zinc-iodine battery by activating the highly reversible I_2_/I^+^ couple (1.83 V vs. Zn/Zn^2+^) in addition to the typical I^−^/I_2_ couple (1.29 V). This is achieved by intensive solvation of the aqueous electrolyte to yield ICl inter-halogens and to suspend its hydrolysis. Experimental characterization and modelling reveal that limited water activity and sufficient free chloride ions in the electrolyte are crucial for the four-electron process. The merits of the electrolyte also afford to stabilize Zn anode, leading to a reliable Zn-I_2_ aqueous battery of 6000 cycles. Owing to high operational voltage and capacity, energy density up to 750 Wh kg^−1^ based on iodine mass was achieved (15–20 wt% iodine in electrode). It pushes the Zn-I_2_ battery to a superior level among these available aqueous batteries.

## Abstract (web summary)

Electrochemically reversible redox couples that embrace more electron transfer at a higher potential are desired for high energy density batteries. Here, the authors report a four-electron aqueous zinc-iodine battery by boosting the iodine electrochemistry in deliberately designed electrolytes.

## Introduction

The ever-increasing demands from portable electronic devices, electric vehicles, and renewable energy sources call for safe, low‐cost storage batteries of high energy density^1^. One approach is to extend state-of-the-art Li-ion batteries to new rocking‐chair systems with earth‐abundant charge carriers, such as Na^+^, K^+^, Ca^2+^, Mg^2+^, Zn^2+^, Al^3+^ ions^2–6^. The potential of using the metal anode instead of the insertion host further promises high energy density. Nonetheless, most of these systems involve organic solvents as the electrolyte, facing intrinsic safety issues in terms of flammability or leakage hazards for large-scale applications. The use of the aqueous solution as the electrolyte, however, has excluded most of the earth-abundant metal anodes. Zinc metal holds a specific capacity of 820 mAh g^−1^, is particularly promising as the anode for the aqueous batteries^7^. Another approach is exploiting multivalent conversion type cathodes, for example, sulfur and oxygen with high specific capacity^8,9^. Though promising, they are challenged by the intrinsic instability of the intermediates product, namely, the polysulfide shuttle and the peroxide/superoxide attack in Li-S and Li/Na-O_2_ batteries^10,11^, respectively. In brief, we believe that developing safe, low-cost and high energy density batteries requires an aqueous system that integrates the metal anode and the multivalent conversion cathode.

The cathode for the aqueous zinc-ion batteries is intensively studied recently, including the intercalation metal oxide frameworks and the conversion type halogens^12^. The metal oxides, mainly the layered MnO_2_^13^ and V_2_O_5_^14^, are plagued by sluggish intercalation/de-intercalation kinetics because of the large electron density of the Zn^2+^ ions thus only limited rate capability and low capacity could be delivered. The halogens, on the other hand, are soluble in electrolyte solvent thus have fast conversion kinetics, as exemplified by the Zn-I_2_^15,16^ and Zn-Br^17^ batteries. It also benefits their applications in organic metal batteries, such as Li-I_2_, Na-I_2_, K-I_2_ to Mg-I_2_, and Al-I_2_ batteries^18–22^. A recent report of a stimulus-responsive Zn–I_2_ battery with overcharge self-protection function achieved by employing a smart pH-responsive electrolyte, solved the substantial safety concern and extends the lifespan of the Zn–I_2_ batteries^23^. These halogen batteries share similar reversible conversion between X^−^ and X_2_/X_3_^−^ (X = Cl, Br and I), however, they fail to fully make the advantages of the high energy density of the metal anodes due to the single electron transfer per halogen ion (or two electrons per halogen). Another recent study managed to intercalate Br° and Cl° into graphite, offering a brilliant concept for designing a high‐energy and safe battery^24^. Previous studies^25–28^ attempt to use high-valence iodine like IO_4_^−^/IO_3_^−^ involving an attractive multi-electrons conversion upon redox in the battery. Although acidic electrolyte and heavy metal salts of the oxy acid of iodine^27,29^ were introduced to enhance the stability of cathode^30^, a reversible battery is yet to be developed. Moreover, the acidic electrolyte decomposes the zinc anode due to severe hydrogen evolution.

In this work, we report a four-electron transfer aqueous zinc-iodine battery by activating the highly reversible I_2_/I^+^ couple in addition to the typical I^−^/I_2_ couple, which doubles the capacity of the conventional iodine batteries. Both experimental characterization and modeling reveal that the new redox couple is correlated to the electrolyte coordination structure: (1) decrease the H_2_O activity of the electrolyte solution by intensive ion coordination to suppress the detrimental hydrolysis of I^+^ species; (2) promote the population of the free chloride ions with higher mobility thus facilitate the I_2_/I^+^ redox kinetics by the formation of ICl inter-halogens. The well-defined two voltage profile of iodine chemistry in the deliberated bisalt electrolyte—holding a theoretical capacity of 422 mAh g^−1^ assume four-electron conversion—delivers a capacity of 594 mAh g^−1^ by combining the double layer capacitor of the carbon host. The benefits of such new iodine chemistry are twofold: (1) the four-electron conversion doubles the capacity been achieved in previous studies involving iodine as the cathode; (2) the reversible conversion of I_2_ ↔ I^+^ occurs at a much higher potential compares to the I_2_ ↔ I^−^ conversion (1.83 vs. 1.29 V, vs. Zn/Zn^2+^), guaranteeing a higher energy density. The energy density of the proposed battery is as high as 750 Wh kg^−1^ based on the iodine mass (495 Wh kg^−1^ based on the total active mass of cathode and anode), which is of great advantage among other sorts of energy system reported before. More importantly, an ultra-long cycle performance of 6000 cycles is achieved, with only 0.003% capacity decay per cycle.

## Results

### Four-electron conversion of iodine in aqueous solution

**Fig. 1:**
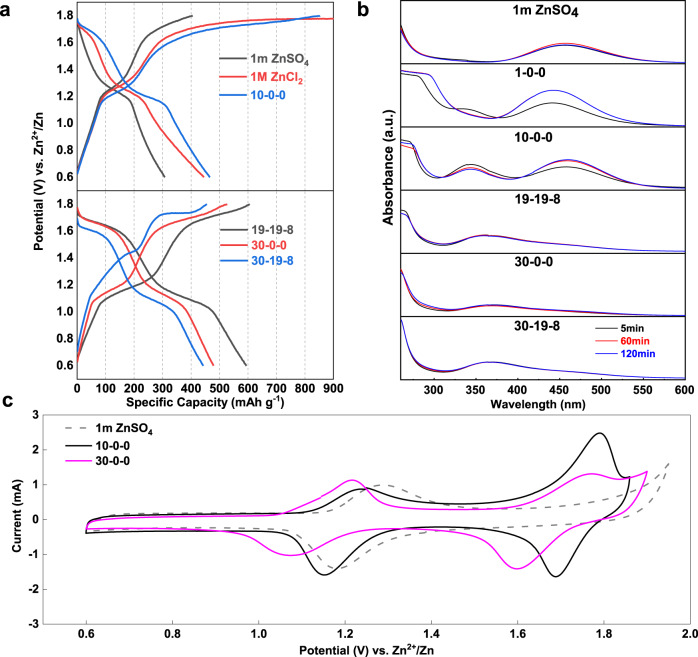
Electrochemical behaviors and UV spectrum characterizations of zinc-iodine battery with different electrolytes. **a**, **b** Charge and discharge voltage profiles of various electrolytes at 400 mA g^−1^ along with UV–vis spectrum of ICl in these solutions recorded with time. **c** Cyclic voltammetry (CV) profiles of selected electrolytes with/without Cl^−^ at 0.5 mV s^−1^.

Simply charge/discharge the iodine electrode (15–20 wt% iodine loaded in PAC carbon) in 1 m ZnSO_4_ solution between 0.6 and 1.6 V vs. Zn/Zn^2+^ shows the typical voltage profile corresponding to the reversible conversion between I^−^ and I_2_, which was well established in the aqueous Zn-I_2_ batteries. A pronounced charge plateau at 1.7 V was arisen when the cut-off voltage was enlarged up to 1.8 V vs. Zn/Zn^2+^ that is far below the oxygen evolution potential (Fig. 1a). This process is ascribed to further oxidation of I_2_, however, no high-order discharge plateau was detected for the subsequent discharge process, indicating the irreversibility of high-valence iodine in the solution, or even unstable in terms of decomposition. The latter is supported by the UV–Vis absorption spectra of the blend solution of commercial ICl and 1 m ZnSO_4_ electrolyte (Fig. 1b), showing a rapid decay of the ICl band between 340 and 360 nm^31^. Meanwhile, the I_2_ peak around 460 nm became dominant. Accordingly, a dark precipitate was detected at the bottom of the solution (Supplementary Fig. 1), indicating the transformation from unstable I^+^ to I_2_ (Note 1 in Supplementary Information). The most straightforward approach to achieve the reversible conversion of the I^+^/I_2_ couple is thus relying on tuning the activity of H_2_O in the electrolyte solution to suspend the hydrolysis.

Early works conducted by Kolthoff and Jordan have systematically studied the I^−^/I_2_ redox processes^32,33^, indicating that under specified conditions both iodide and iodine yield an anodic wave corresponding to the formation of positive iodine (I^+^). Further studies^34,35^ concluded that IO_3_^−^ was the direct oxidation product of iodine in the absence of halide (Cl^−^, Br^−^) or cyanide (CN^−^) in an aqueous solution. This is due to the interaction between electrophilic I^+^ and nucleophilic species like halide^36^, cyanide^32^, and amines^37^ which afford to charge-transfer complexes^38^.

ZnCl_2_ was chosen to coordinate with H_2_O molecule so as to suppress the I^+^ cation hydrolysis due to three reasons: (1) high solubility in water (up to 31 m, molality); (2) the redox potential of Cl_2_/Cl^−^ is high enough (1.396 V vs. SHE) to tolerate I^−^/I_2_/I^+^ conversion than that of Br_2_/Br^−^(1.087 V)^33^; (3) the nucleophilic ability of Cl^−^ is relatively strong enough to form stable but of redox activity ICl compared with less stable IBr or non-electrochemical ICN. LiCl was introduced into ZnCl_2_ solution to provide extra Cl^−^ source to the parental solution. It possesses high solubility and miscibility with ZnCl_2_ compared with its analogous alkali halides, along with the specific solvation nature of lithium cation, could further regulate the coordination between Zn^2+^, H_2_O, and Cl^−^. Acetonitrile (ACN), one of the most oxidative resistance solvents (above 4.8 V vs. Li^+^/Li), is acted as a diluent to facilitate ion mobility. Moreover, ACN is inert to I^+^ which is obviously of great advantage in our system. The electrolyte formula we defined is the blends of specific molality of ZnCl_2_, LiCl, and ACN in water (donated as *x*-*y*-*z*). Extensive ion coordination in the concentrated electrolyte pushes the electrochemical window of the aqueous electrolyte to higher potentials (Supplementary Fig. 2).

The UV-Vis absorption spectra of the less concentrated electrolytes (1-0-0, 10-0-0) resemble the tendency of I^+^ decomposition in the 1 m ZnSO_4_ solution (Fig. 1b). In contrast, enhanced capability to stabilize ICl could be achieved in concentrated systems (19-19-8, 30-19-8, 30-0-0). Little intensity changes of the ICl band was observed in the whole duration without the appearance of the I_2_ signal.

The correlation of the electrolyte formula and the iodine redox chemistry were conveyed by galvanostatic charge/discharge (GCD). Distinctive discharge plateaus at 1.75 V (absent in the 1 m ZnSO_4_ electrolyte) are observed in the chloride-containing electrolyte even in the diluted 1 m ZnCl_2_ solution (Fig. 1a). Though it was still plugged by I^+^ ion hydrolysis as reflected by the low reversibility, we ascribe it to the nucleophilic Cl^−^ which provides affinity to the I^+^ ions. It is obvious that the high-order discharge plateau became pronounced with the increase of the ZnCl_2_ concentration, indicating suppressed I^+^ ion hydrolysis. The 30 m ZnCl_2_ electrolyte delivers a specific capacity of 550 mAh g^−1^ and the 19-19-8 hybrid electrolyte reveals a striking reversible capacity as high as 594 mAh g^−1^ at 400 mA g^−1^ (Fig. 1a). Note that these capacities are beyond the theoretical capacity of 422 mAh g^−1^ based on the I^+^ ↔ I_2_ ↔ I^−^conversion. We ascribe it to the capacitance behavior of the active carbon host^39,40^, which shows a specific capacity of ~38 mAh g^−1^ based on the carbon weight (or ~180 mAh g^−1^ based on the iodine mass) with typical capacitance response in the electrolytes (Supplementary Fig. 3). This is in accordance with the contribution of porous carbons in the conventional Zn-I_2_ batteries^41^. The CV curves in these electrolytes are shown in Fig. 1c and Supplementary Fig. 4. The 1 m ZnSO_4_ electrolyte presents only one reversible redox peak in the absence of Cl^−^, showing the I^−^/I_2_ conversion around 1.28 V/1.19 V. However, the introduction of Cl^−^ introduces an additional redox couple appearing at 1.75/1.65 V. Such new couple is in accordance with previous reports^33^, which is attributed to the redox of I_2_/I^+^.

**Fig. 2:**
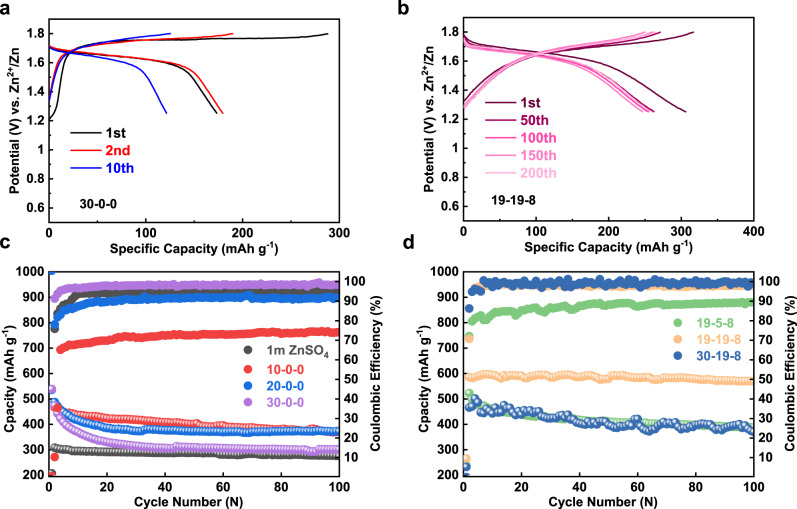
Electrochemical performances iodine electrode in various electrolytes. **a**, **b** Charge and discharge voltage profiles of the 30-0-0 and 19-19-8 electrolyte from 1.25 to 1.8 V at 400 mA g^−1^, respectively. **c**, **d** Full-range (0.6–1.8 V) cycling performance of different systems at 400 mA g^−1^.

Comparison is made between the single salt electrolyte (30-0-0) and the hybrid electrolyte (19-19-8), showing that the hybrid electrolyte delivers a higher discharge capacity with nearly equal contribution from the high-order and the low-order plateau (Fig. 1a). It is apparent that the total specific capacity of the iodine electrode between 0.6 and 1.8 V (I^+^ ↔ I^−^) is determined by I^+^ ↔ I_2_ conversion, which is governed by the tendency of I^+^ ion hydrolysis. We tested the iodine electrode between 1.25 and 1.8 V with only the I_2_/I^+^ conversion being activated (Fig. 2a, b). The hybrid 19-19-8 electrolyte possesses more stable capacity retention than 30-0-0 and other dilute electrolytes. The cycle performance of pure ZnCl_2_ solution with different molality has been revisited as well (Fig. 2c). While the coulombic efficiency is in direct proportion to concentration, the 30 m ZnCl_2_ system showing minimal hydrolysis of I^+^ is still suffered severe capacity decay. We attribute this unsatisfactory performance to the formation of non-electroactive Zn-I clusters during cycling(Supplementary Fig. 5), which is in accordance with the phenomenon reported by Ji et al.^42^.

Apart from an abnormal potential curve with a significant voltage gap between charge/discharge plateau in 30-19-8 electrolyte, the distinct fluctuation of Capacity-CE profile is attributed to its high viscosity (Figs. 1c, 2d). On the other hand, the 19-5-8 system only exhibits a low coulombic efficiency, which is analogous to the 20 m pure ZnCl_2_ due to a conspicuous degree of hydrolysis (Fig. 2c, d). We further quantified the self-discharge by testing the retained capacity after storage of a charged Zn-I_2_ cell at open-circuit voltage (OCV) (Supplementary Fig. 6). There was significant capacity decay with progressive OCV fading for the 19-5-8 cell, whereas both 30-19-8 and 19-19-8 cells preserve high capacity and high OCV after the storage. Supplementary Fig. 6e confirms that the capacity loss during the intervals (self-discharge) in the 19-19-8 electrolyte could be recovered in the following cycles. These electrochemical results are in good agreement with the UV–vis spectrum discussed in Fig. 1. We note the proposed four-electron transfer Zn-I_2_ battery could also be started from the discharged state with dissolved ZnI_2_ in the electrolyte, as demonstrated by 0.1 M in the 30-0-0 electrolyte (Supplementary Fig. 7). Only a blank carbon electrode was used as the cathode in this case. The lower coulumbic efficiency of the Zn-ZnI_2_ battery than that of the Zn-I_2_ battery emphasizes the importance of physical confinement of the preloaded iodine by PAC hosts.

It is apparent that a suitable concentration is required to allow the normal operation of the battery to suppress the self-discharge reaction. We speculate the enhanced stability towards I^+^ species is correlated to the suppressed water activity and the sufficient chloride ions in the concentrated solution, which will be further discussed in the next section.

### Understanding the chemistry of the four-electron conversion

**Fig. 3:**
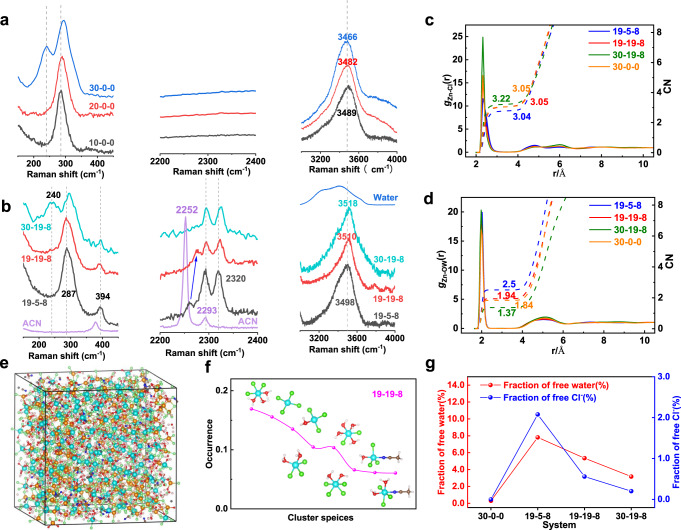
MD simulation and Raman spectrum characterization on the properties of electrolytes. **a**, **b** Raman spectra of solutions of different concentrations. **c**, **d** Radical distribution function(RDF) of the solutions. The solid lines are the radial distribution functions, and dotted lines are the coordination numbers. **e** A snapshot of the MD simulation box for 19-19-8 system, along with the dominant clusters existing in such system (**f**). Clusters and their occurrence in the 19-19-8 system. Cyan-blue atom is Zn^2+^, green atom is Cl^−^, yellow atom is Li^+^, red atom is O, dark blue atom is N, brown atom is C, gray atom is Me, and white atom is H. **g** Summary of free water and Cl^−^ content extracted from the MD simulation.

Raman spectroscopy combined with molecular dynamics simulations (MD) were conducted to get insight into the solution structure and solvation environment of the electrolytes, for the purpose of illustrating the deep distinctions of their electrochemical performance. The main response region of the Zn-Cl vibration located from 150 to 450 cm^−1^ is shown in Fig. 3a, b. The intense Raman band at around 290 cm^−1^ represents the signal of the Cl associated Zn hydrates [ZnCl_*2+x*_(H_2_O)_*y*_]^*x−*^, existing in all solutions surveyed^43,44^. With increasing the content of chloride salt, another peak at 240 cm^−1^ emerged which is attributed to the tetra-coordinated ZnCl_4_^2−^ cluster in 30-0-0 and 30-19-8 solution, suggesting a large portion of Zn^2+^ ions were surrounded tightly by Cl^−^ due to insufficient water molecules around the cations that forming the hydration shells. The ZnCl_4_^2−^ clusters are absent in other solutions with less salt concentrations.

The ACN solvent has two peaks appearing at 2252 and 2293 cm^−1^ which is assigned to ν(CN) stretching vibration and a combination band, respectively^45^. New peaks are readily observed at 394 and 2320 cm^−1^ when ACN was introduced in ZnCl_2_ solutions, corresponding to the ν(CN) stretching mode of the ACN bound with Zn^2+^/Li^+^ ions in the first coordination sphere^46,47^. In the meanwhile, the peak of free ACN shows a blue-shift to 2263 cm^−1^ in 19-5-8 and further shifts to 2276 cm^−1^ in 19-19-8. Such phenomenon is attributed to the interaction between ACN and the coordinated water molecules—the enlarged content of those water molecules solvating with cation ion results in a shift of the ACN to a higher frequency^47,48^. These phenomena are in accordance with the characteristic C-C ν4 band of ACN (918 cm^−1^, Supplementary Fig. 8). A much distinguished blue-shift observed in 19-19-8 compared with 19-5-8 further renders a higher ratio of coordinated water in the solution. The ν(CN) stretching vibration faded away successively, and finally vanished in 30-19-8 solution^45^. We summarized the free ACN fraction in these bi-salt electrolytes by MD simulation (Supplementary Fig. 9), based on the coordination numbers at the first solvation shell of the Zn^2+^ and Li^+^ ions (Supplementary Fig. 10). The limited occurrence number of acetonitrile around cations is mostly because of its preferential solvation by water and anions. The high ratio free ACN molecules is in accordance with our original concept, which was designed to decrease the viscosity and to promote ion conduction.

MD simulations afford more detailed structural information of the electrolytes (19-5-8, 19-19-8, 30-19-8, and 30-0-0, see the method in supporting information). Information about the structural properties of the environment around an ion (cation or anion) is determined from the radial distribution functions (RDF, *g*_αβ_(*r*)) and the corresponding average coordination numbers in the first coordination sphere. The significance of Zn-Cl and Zn-O RDF, determined by the magnitude of peaks, indicating strong ion association and ion hydration mostly took place around the Zn^2+^ ions (Fig. 3c, d). Other cases of coordination are shown and discussed in Supplementary Fig. 10 for clarity. The Zn-Cl association is enhanced with the increase of the salt concentration, whereas the hydration is weakened at a high concentration. This, combined with the varies of the coordination number in the nearest shell, indicating a possible transformation of hydrated Zn^2+^ ion to Zn-Cl association at high concentrations. The transformation is attributed to the higher chloride ion population in the solution at high salt concentrations, which is demonstrated by the occurrence of the overall average coordination numbers, and the respective Zn-*x*Cl and Zn-*y*H_2_O coordination analysis (Supplementary Fig. 11). Indeed, the salt concentration has a significant impact on the cluster structure and its occurrence in the electrolyte, as shown by the simulation boxes and the corresponding distribution occurrence of the clusters (Fig. 3e, f, and Supplementary Fig. 12). High-concentration chloride ions repel H_2_O molecules from the hydration shell, resulting in clusters with less hydrated structure, namely, the most dominant [ZnCl_3_(H_2_O)_3_]^−^ clusters in 19-19-8 solution vs. [ZnCl_3_(H_2_O)]^−^ or even clusters without hydration [ZnCl_4_]^2−^ in 30-0-0 and 30-19-8 solutions. This is consistent with the RDF analysis and their corresponding coordination number, and also obeys to the Raman results. However, the lower occurrence of hydrated cluster structure does not imply a high fraction of free H_2_O in solutions with high salt ratios, as shown Fig. 3g. Adversely, the free water decreases with the increase of the salt concentration. It is attributed to more cations presented in the concentrated solution that widely harvest the water molecules, which is also reflected by the overall Zn-yH_2_O coordination analysis (Supplementary Fig. 11). The free chloride ion in different solution has a similar tendency as the free H_2_O, with the 19-5-8 solution has the highest free Cl^−^ ions ratio (2%). This is owing to the globally formation of Zn-Cl···Zn-Cl and Li-Cl···Li-Cl polymeric chain in high-concentration solutions (Fig. 3e, and Supplementary Fig. 12), which suppresses the chloride ion exchange between the bulk solution and coordinated clusters. Notably, the 19-19-8 solution has moderate fraction of free chloride ion (0.6%) and free H_2_O (5%) simultaneously.

Supplementary Fig. 13 shows the physicochemical properties of viscosity and ionic conductivity. In pure ZnCl_2_ solutions, 30-0-0 owns the highest viscosity (397 cp) and lowest ionic conductivity (8.14 mS/cm). The tightly coordinated ZnCl_4_^2−^ cluster ascribes the large viscosity and the low ionic conductivity of the concentrated solution. Taking water in bisalt system into consideration, similar properties can be observed in 30-19-8 (339 cp and 14.5 mS/cm, respectively). However, the ionic conductivity of 19-19-8 does not bear any compromise compared with 19-5-8, even in the same level of 20-0-0, which is benefited from its relatively low viscosity (75.8 cp). The ionic conductivity of the 19-19-8 electrolyte is five times lower than that of the 1 M ZnSO_4_ electrolyte (28 vs. 145 mS/cm), but it is sufficient for battery application.

We conclude that in dilute ZnCl_2_ solutions (10-0-0, 20-0-0, and 19-5-8), high water activity rendering rapid hydrolysis of ICl, which results in low coulombic efficiency as discussed in Fig. 2. Whereas in solutions with very high concentrations (30-0-0 and 30-19-8), the low Cl^−^activity and the substantial coordination of iodide with Zn^2+^ (Zn-I clusters, Supplementary Fig. 5) is attributed to the rapid capacity decay. Only the 19-19-8 solution takes full advantages of low fraction of free water to suppress I^+^ hydrolysis and sufficient ionic conductivity with high Cl^−^ activity to realize good performance.

**Fig. 4:**
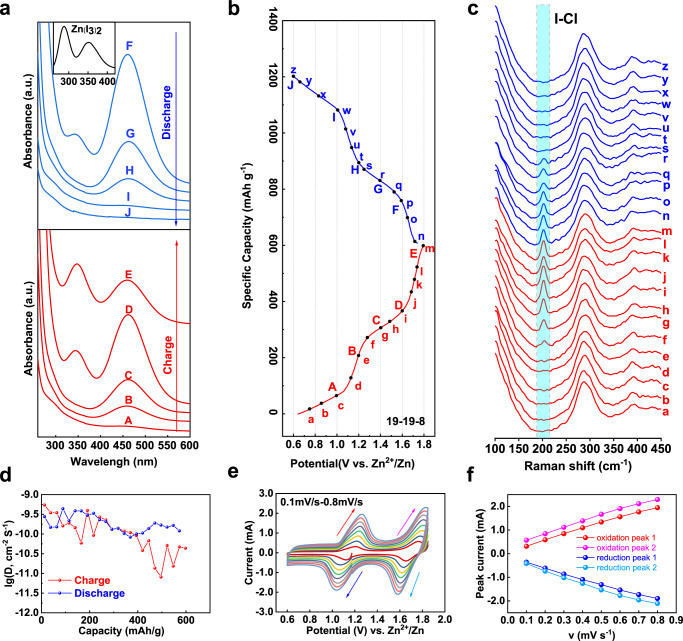
Spectrum and electrochemistry characterizations to reveal the mechanism of the four-electron conversion process. **a** Ex situ UV vis spectrum of 19-19-8 system recorded at different charge-discharge stages. **b** Typical voltage-capacity profile of 19-19-8. **c** In situ Raman spectrum of 19-19-8 system during charge/discharge. **d** Diffusion coefficient versus specific capacity, calculated from GITT measurements. **e** CV curves with different scanning rates from 0.1 to 0.8 mV s^−1^. **f** The plots of the oxidation and reduction peak-current with the function of the root of scanning rates.

To illustrate the involved four-electron reaction mechanism, ex situ UV–vis spectrum associated with in situ Raman spectroscopy were conducted to verify the electrode species and its stability during charge/discharge (Fig. 4). Figure 4a, b convey the ex situ UV–vis spectrum in the course of charge/discharge process in 19-19-8 electrolyte. An adsorption peak arising at 460 nm was attributed to the formation of iodine molecules when charge to 1.2 V, which confirms the conversion of iodide to iodine for the first charge plateau. It is noteworthy that, in contrast to the conventional organic metal-iodine battery, no peak of triiodide I_3_^−^ was detected in the whole duration (see inserted spectrum)^41^, suggesting a direct conversion of −1 and 0 valence iodine species. Further charge of the electrode generates another adsorption peak at around 342 nm, in analogous to the previous reports^49^, which is assigned to the formation of ICl inter-halogens.

We evaluated the interaction of iodide (discharge state) with the electrolyte by MD simulations. The significance of Zn-I coordination of the 30-0-0 solution is much stronger than the 19-19-8 system, as determined by the magnitude of the first coordination shell (RDF results, Supplementary Fig. 14). This indicates iodide ion could participate in cation coordination in 30-0-0 system (contact ion pair, CIP), which might drag the iodide diffuse from the electrode. However, the iodide and zinc coordinated as a solvent-separated ion pair (SSIP, indirect interaction) in the 19-19-8 solution. The CIP coordination of zinc-iodide might attribute to the formation of the non-electroactive ZnI_2_(OH_2_)_2_ clusters in the 30-0-0 electrolyte (Supplementary Fig. 5), which leads to capacity fading in the battery.

Real-time transformation of the iodine species was monitored by in situ Raman spectroscopy within a Raman electrolytic device (Supplementary Fig. 15, Fig. 4c). The major peak detected at 288 cm^−1^ among the whole course is assigned to [ZnCl_*2+x*_(H_2_O)_*y*_]^2−*x*^ clusters^43^, which again demonstrates the main coordination state of the electrolyte solution. The new Raman signal emerged around 202 cm^−1^ when charge to 1.4 V is corresponding to the characteristic band of ICl species^50,51^. The iodine intermediate was proved to form during the first charge plateau by the ex situ UV–vis spectrum. However, its Raman signal is failure to be detected under such experimental parameters, even absent in 1 m ZnSO_4_ solution (Supplementary Fig. 15), which is in consistent with previous reports^41^. During discharge, the ICl peak faded away with the discharge depth and vanished eventually at the end of high-order plateau, again indicating a reversible I^+^/I_2_ conversion. In situ Raman characterization of other systems (1 m ZnSO_4_ and 30 m ZnCl_2_, Supplementary Fig. 16) show identical iodine speciation in the 30 m ZnCl_2_ solution but only I_2_/I^−^ conversion occurred in 1 m ZnSO_4_ solution. Note that none Raman signal of Zn(I_3_)_2_ was found neither in concentrated electrolytes (30 m ZnCl_2_) nor in 1 m ZnSO_4_ system.

Based on the electrochemical analysis and spectral results, we depict a clear portrait of the reaction mechanism for the four-electron conversion:

Cathode:

1 $$2{\mathrm{I}}^{-} \leftrightarrow {\mathrm{I}}_2 + 2\,{\mathrm{e}}^ -\, {\mathrm{E}}^{\mathrm{o}} = 0.53{\mathrm{V}}\,{\mathrm{vs}}.\,{\mathrm{SHE}}$$

2 $${\mathrm{I}}_2 + 2\,{\mathrm{Cl}}^ - \leftrightarrow 2\,{\mathrm{ICl}} + 2\,{\mathrm{e}}^ -\, {\mathrm{E}}^{\mathrm{o}} = 1.07{\mathrm{V}}\,{\mathrm{vs}}.\,{\mathrm{SHE}}$$

Anode:

3 $${\mathrm{Zn}}^{2 + } + 2\,{\mathrm{e}}^ - \leftrightarrow {\mathrm{Zn}}\,{\mathrm{E}}^{\mathrm{o}} = - 0.76{\mathrm{V}}\,{\mathrm{vs}}.\,{\mathrm{SHE}}$$

Total reaction:

4 $${\mathrm{Zn}}^{2 + } + 2\,{\mathrm{I}}^ - \leftrightarrow {\mathrm{Zn}} + {\mathrm{I}}_2\,{\mathrm{E}} = 1.29{\mathrm{V}}$$

5 $${\mathrm{Zn}}^{2 + } + {\mathrm{I}}_2 + 2\,{\mathrm{Cl}}^ - \leftrightarrow {\mathrm{Zn}} + 2\,{\mathrm{ICl}}\,{\mathrm{E}} = 1.83{\mathrm{V}}$$

Galvanostatic intermittent titration technique (GITT) was used to study the kinetics of reactions (Supplementary Fig. 17, see method for details). The quasi-equilibrium potentials are about 1.18 V for I^−^/I_2_ and about 1.71 V for I_2_/ICl oxidation, respectively. The diffusion coefficient yielded from GITT is in the order of 10^−9^–10^−10^ cm^2^ S^−1^ (Fig. 4d). CV curves of the 19-19-8 system with different sweep rates from 0.1 to 0.8 mV s^−1^ are shown in Fig. 4e. The linearly increased oxidation and reduction peak-current (*i*) as a function of the root of scan rates (*ν*^1/2^), indicates both I^−^/I_2_ and I_2_/I^+^ conversions are diffusion-controlled redox reactions (Fig. 4f). Moreover, kinetic analyzing adopts from the log *i*—log *ν* plots (Supplementary Fig. 18) show the slope of the peaks at lower potential is normally higher than that at the higher potential (either reduction or oxidation), indicating the conversion of I^−^/I_2_ is faster than the I_2_/I^+^ redox couple.

### A high energy density aqueous Zn-I_2_ battery based on four-electron conversion

The electrolyte formula (19-19-8) we proposed has the advantage of suppressed H_2_O activity and preserved free chloride ions, which facilitate the four-electron conversion of iodine with a high reversible capacity. While a practical battery relies on the robust anode as well, we tested the zinc anode in the 19-19-8 electrolyte to elucidate the electroplating behavior.

**Fig. 5:**
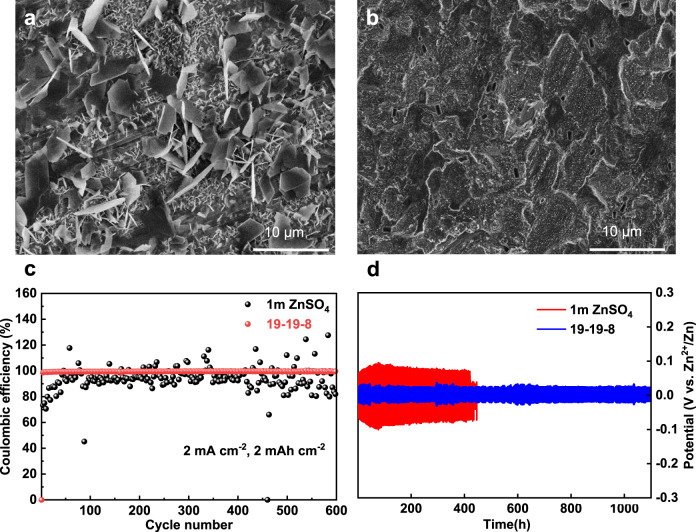
Characterization of the Zn metal anode in the electrolyte. **a**, **b** SEM images of the Zn electrodes cycled for 1 h at 1 mA cm^−2^ in 1 m ZnSO_4_ and 19-19-8, respectively. **c** Coulombic efficiencies of zinc plating/stripping on Ti foil (2 mA cm^−2^, 1 h for the plating). **d** The plating/stripping in Zn||Zn symmetric cells at 1 mA cm^−2^ with a sweep duration of 10 min.

It is apparent that zinc anode suffers serious dendrite formation in normal aqueous solution (1 m ZnSO_4_), as shown in Fig. 5a. The plated zinc anode shows two series of morphologies, namely polygons plates and clusters both with sharp edges. The disadvantages of these dendrites are twofold: they might pierce through the separator leading to a short-circuit and the high surface area dendrite could result in detrimental side reaction with electrolyte. The latter is illustrated by the deteriorative coulumbic efficiency of the Zn||Ti battery in the 1 m ZnSO_4_ electrolyte (Fig. 5c). In sharp contrast, the zinc plated in the 19-19-8 electrolyte shows dense and smooth morphology with the same test condition (Fig. 5b). The morphology of the zinc anode after long-term cycling further confirms the favorable electrodeposition in the 19-19-8 electrolyte (Supplementary Fig. 19). The less dendritic deposition is attributed to the aggregated ion density at high ZnCl_2_ concentration suppressing the ion-depletion, which is regarded as the main reason for dendrite nucleation^43^. Plating/stripping in Zn‖Zn symmetric cells (1 mA cm^−2^, 1 h) further demonstrates the robust of the zinc anode in the 19-19-8 electrolyte (Fig. 5d). The 19-19-8 electrolyte sustains highly stable voltage profile over 1100 hours without any significant overpotential fluctuation, whereas the 1 m ZnSO_4_ electrolyte shows higher overpotential and the cell is only stable for about 400 h. Moreover, the Zn‖Ti battery in the 19-19-8 electrolyte shows a coulumbic efficiency of 99.8% for over 600 cycles (Fig. 5c). The highly reversibility of the zinc anode in the 19-19-8 electrolyte is benefited from the suppressed H_2_O activity that mitigates the formation of Zn(OH)_2_/ZnO^43^.

**Fig. 6:**
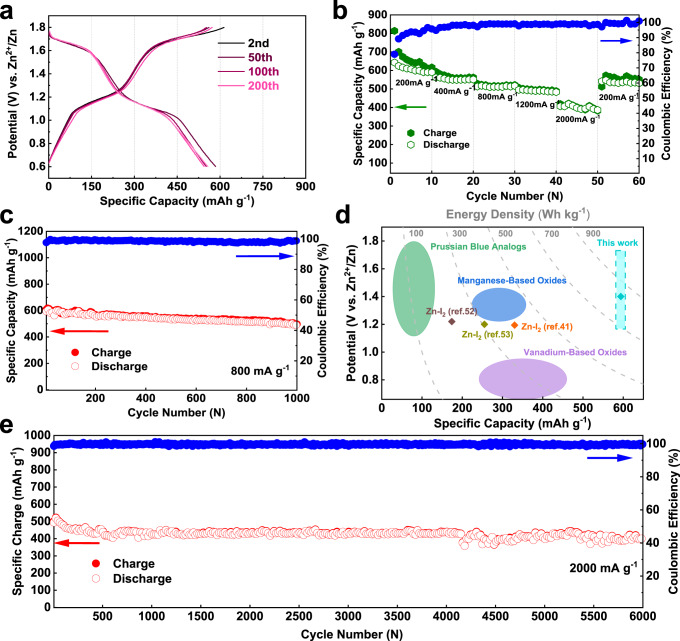
The electrochemical performance of 19-19-8. **a** Comparative voltage profiles at 400 mA g^−1^ for 200 cycles. **b** Rate test at different current densities. **c** Cycling performance at 800 mA g^−1^ for 1000 cycles. **d** The energy density, average voltage, and specific capacity of aqueous zinc batteries based on intercalation chemistry and conversion chemistry. For comparison, energy densities were converted with active materials only. The intercalation Zn-ion batteries were quoted from ref. ^12^. **e** Long-term cycling at a high rate of 2000 mA g^−1^ for over 6000 cycles.

The cycling performance of the zinc iodine battery was performed with iodine loaded active carbon electrode. Iodine was loaded onto active carbons by physical adsorption in water, as confirmed by the mass changes and the surface area variations of the composite (Supplementary Fig. 20). EDS mapping reveals that iodine is homogenously distributed on the electrode (Supplementary Fig. 21). XPS characterization of the sample shows a splitting of the core-level spectra of I_3d_, indicating electron transformation between iodine and the carbon matrix (Supplementary Fig. 22)^52^. Given the fact the Zn-I_2_ battery was assembled at the middle valance state of iodine, we confirmed that there is little difference for the cell that being activated by charge or by discharge (Supplementary Fig. 23). Supplementary Fig. 24 shows the cycling performance of the Zn-I_2_ battery in a flooded electrolyte condition (80, 160 μL), demonstrating similar performance as the 50 μL cells. A slight capacity fading is observed from the charge/discharge curves at a low current density of 400 mA g^−1^ for over 200 cycles; and a coulombic efficiency of 98.5% was obtained (Fig. 6a). Rate capability was examined from 200, 400, 800, 1200 to 2000 mA g^−1^, showing high reversible capacities of 609, 589, 530, 507, 419 mAh g^−1^, respectively (Fig. 6b). The coulumbic efficiencies at these rates are very high, rendering controlled I^+^ hydrolysis (self-discharge). Long cycling performance at a moderate rate of 800 mA g^−1^ (around 90 min per cycle) indicated a very promising capacity retention at 85.4% after 1000 cycles (Fig. 6c). The reliability of four-electron conversion was also examined at a remarkable high current density of 2000 mA g^−1^, revealing a reversible capacity of 420 mAh g^−1^ with a superior longevity as long as 6000 cycles, corresponding to a capacity decay rate of 0.003% per cycle (Fig. 6e). The remarkable long lifespan is attributed to the highly reversible I^−^/I_2_ and I_2_/I^+^ redox couples, while the suppressed solubility of the iodine species by the concentrated electrolyte and the physical confinement provided by the PAC carbon further ensure high utilization of iodine^52^. Aside from that, the stable plating/stripping of Zn anode also benefit to such notable battery performance.

Note that the formation of ICl inter-halogens was not necessarily limited by the content of free Cl^−^ ions in the electrolyte. This was supported by the formation of ICl in the 30-0-0 electrolyte (Supplementary Fig. 16), which shows none free Cl^−^ ions according to the MD simulation. We developed doped carbon to substitute the PAC carbon as the iodine host. Due to the porous structure and the affinity towards iodine species by the heteroatoms, high iodine loading (~45 wt% in the composite, and 9 mg cm^−2^ in the electrode) could be achieved. This electrode has similar cycling performance as the I_2_/PAC electrode with the identical condition (Supplementary Fig. 25), again indicating that the four-electron redox chemistry is not simply limited by the free fraction of Cl^−^ ion in the electrolyte. However, too high iodine mass in the cell might result in the conjugation of the electrolyte quantity and the iodine mass. This is due to the significant electrolyte diluting caused by ion depletion during the charge process at high iodine loading, which releases of free water to a large extent (triggers ICl hydrolysis) thus limit the reversibility.

For comparison, we plotted our four-electron conversion Zn-I_2_ battery in Fig. 6d against other aqueous systems. The high discharge plateau of the I_2_/I^+^ redox couple increases the average operational voltage of the Zn-I_2_ battery (1.45 V vs. 1.2 V of the conventional Zn-I_2_ batteries), affording to a high energy density of 750 Wh kg^−1^ (base on the iodine mass). This gives a sharp demonstration of the merits of our system over typical zinc-iodine batteries^41,52,53^, and holds a distinct place among intercalation electrodes for aqueous zinc-ion battery^12^ and state-of-the-art aqueous Li/Na-ion batteries^54^. The full-cell energy density after considering the anode mass still reaches 495 Wh kg^−1^, which is at the same level of the recent reported halogen intercalated graphite Li-ion battery^24^. The Ragone plots derived from the rate performance further illustrate the high energy density as well as the high power density of the four-electron conversion Zn-I_2_ battery (Supplementary Fig. 26), indicating promising applications by considering the intrinsic safety brought by its aqueous nature. Noting high iodine loading and high iodine utilization could be achieved by developing new host materials that are highly conductive and provides affinity to the iodine species during the electrochemical process.

## Discussion

In summary, we have developed an aqueous zinc-iodine battery with four-electron transfer per iodine by activating the I_2_/I^+^ couple (1.83 V *vs*. Zn/Zn^2+^) in addition to the I^−^/I_2_ couple (1.29 V), which doubles the capacity of the conventional iodine batteries either in organic or in aqueous media. This is correlated to the dynamics of water and chloride ions in the electrolyte solution, as demonstrated by both spectroscopy studies and modeling, showing the molality controlled coordination behavior fully manipulates the ICl inter-halogens formation and its hydrolysis. The extended iodine chemistry in the deliberated bisalt electrolyte (19-19-8) doubles the capacity that was achieved in conventional iodine batteries, further boosting the energy density of the developed Zn-I_2_ batteries due to the distinctive high potential. The energy density of the proposed battery is as high as 750 Wh kg^−1^ based on the active materials mass of cathode (iodine fraction in the cathode is 15–20 wt%). More importantly, an ultra-long cycle performance of 6000 cycles is achieved with only 0.003% capacity decay per cycle. Such rechargeable Zn-I_2_ batteries have the potential to be cost effective and safe, and to have high power density for practical application. Further studies on other types of carbonaceous or non-carbonaceous materials with stronger interaction with iodine are highly desired to ensure a high utilization of the active material. Moreover, optimizing the amount of electrolyte and the capacity matching between the cathode and anode are crucial in realizing a high energy density of the four-electron Zn-I_2_ practical full battery.

## Methods

### Preparation of PAC-I_2_ cathodes

Solution-adsorption method was used for the preparation of cathodes. In brief, 300 mg of I_2_ were mixed with 300 mg PAC, followed by adding 20 ml deionized water. The Iodine ratio in the I_2_/PAC composite was about 15–20 wt%, as calculated by deducting the mass of PAC from the final weighted composite. The PAC/I_2_, sodium carboxymethyl cellulose (CMC) and super P were mixed in deionized water with a mass ratio of 8:1:1. Then the slurry was cast on a titanium foil followed by drying for 12 h in the air at 60 °C. The electrodes were cut into disks with a diameter of 10 mm. The average areal loading of PAC-I_2_ composite was about 4–5 mg cm^−2^ in the electrode.

### Materials characterization

Raman analyses were carried out on a bench Raman dispersive microspectrometer (InVia Reflex, Renishaw) using a laser (wavelength of 532 nm) at frequencies from 100 to 4000 cm^−1^. Raman electrolytic device was implemented for the in situ Raman test, the cathode slurry (PAC/I_2_ with binder) loaded at the bottom of the cell (namely, glass carbon electrode) was dried at 60 °C, which is served as the working electrode. The counter electrode is Pt wire and the reference electrode is Ag/AgCl, respectively. The cell was sealed with silica window. The galvanostatical charge/discharge was conducted on a Bio-logic electrochemical station (VSP) with a current density of 0.05 mA cm^2^. UV–vis spectrum characterization was carried out on a UV1902PC with a range from 200 to 600 nm. XRD measurements were carried out on a Bruker D8-Advance powder X-ray diffractometer operating at 40 kV and 30 mA, using Cu-Kα radiation (*λ* = 0.15405 nm). SEM studies were carried out on a FEI QuANTA 200 environmental SEM instrument and a JSM-6700F field-emission SEM instrument. Electrodes were gently washed with deionized water to remove the electrolyte and dried for 12 h in air at 60 °C prior to the characterizations. Brunauer-Emmett-Teller (BET) measurements were carried out on a JW-BK200C surface area analyzer operating at nitrogen atmosphere and the adsorption temperature of 77 k. Samples were dried for 12 h in air at 60 °C prior to the characterization.

### Electrochemical measurements

Electrochemical studies were performed in PFA-based Swagelok-type cells (1/2-inch diameter). Zinc foil with 0.1-mm thickness was used as the anodes. Glass fiber was placed between the anode and cathode as the separator. The electrolyte amount for each cell was 50 μL. The cells were then galvanostatically tested on a Land BT2001A battery test system (Wuhan, China) at room temperature. For the Zn∥ Zn symmetric cells, we used a protocol of 1 hour of stripping followed by 1 h of plating with a current density of 1 mA cm^−1^ for each cycle. Cyclic voltammetry measurements and the GITT tests were performed on a VSP-3 electrochemical workstation (Bio-Logic, France) in a three-electrode cell with the Ag/AgCl reference electrode. The scanning voltage range was set from −0.4 to 0.9 V at scan rates various from 0.1 to 0.8 mV s^−1^. The GITT test, consisting of a series of current pulses (≈400 mA g^−1^) for 1 min followed by a 10 min relaxation process, was performed with the voltage range from 0.6 V to 1.8 V (vs Zn^2+^/Zn).

### Computation

Molecular dynamics simulations of four different high-concentration mixed systems were carried out with the DL_POLY_4.06 software package^55,56^ to investigate the effects of microscopic hydration characteristics on battery performance. The system mainly consists of ZnCl_2_, LiCl, H_2_O or CH_3_CN. The molar ratios of ZnCl_2_: LiCl: CH_3_CN are 19-5-8, 19-19-8, 30-19-8, and 30-0-0, respectively, keeping the molar concentration of water unchanged. In the entire simulation system, the density of the box is maintained at ~1 g/cm^3^. Details of the simulation boxes of the four systems are shown in Supplementary Tables 1 and 2 and Supplementary Note 2.

## Supplementary information

Supplementary Information

## Data Availability

The authors declare that all the relevant data are available within the paper and its Supplementary Information file or from the corresponding author upon reasonable request.
