# Supplementary material for: A four-electron Zn-I2 aqueous battery enabled by reversible I−/I2/I+ conversion
Source: Nat Commun. 2021 Jan 8;12:170. doi: 10.1038/s41467-020-20331-9 (PMC7794333; doi:10.1038/s41467-020-20331-9)
Supplement: Supplementary file 1 — Supplementary Information [file 41467_2020_20331_MOESM1_ESM.pdf]

Supplementary Information

Supplementary Note 1

Early works conducted by Kolthoff and Jordan have systematically studied the I^-/I_2 redox processes[1,2], indicating that under specified conditions both iodide and iodine yield an anodic wave corresponding to the formation of monovalent iodine cation(I^+). Further studies[3,4,5] concluded the direct oxidation products of iodine are charge-transfer complexes with halide (Cl^-, Br^-) or cyanide (CN^-) in aqueous solution. This is due to the strong interaction between electrophilic I^+ and nucleophilic species like halide[6], cyanide[1] and amines[7]. However, the chemical stability of these charge transfer complexes have to be addressed against from hydrolysis in the aqueous media.[6,8,9] Taking ICl as an example, it is inevitable that ICl would be hydrated by water to form H_2OICl. This is owing to the unsaturated coordination sphere of iodine in ICl, while the oxygen atom of water molecule can donate its lone-pair electrons to form the hydrate complex.[8, 10] The deprotonation or the disproportionation of H_2OICl hydrate yields HIO, *via.* $H_2OICl \leftrightarrow HOI + HCl$, or $H_2OICl \leftrightarrow H_2OI^+ + Cl^- \leftrightarrow HOI + HCl$, as summarized in **Eqn. 1.** The manner of the decomposition of hypoiodous acid HIO is much influenced by the concentration, pH and temperature of the solution, by photolysis and by metal catalyst (**Eqn. 2 and 3**).

$$ICl + H_2O \rightleftharpoons HIO + HCl \qquad \textbf{(1)}$$

$$4HIO \rightleftharpoons 2I_2 + 2H_2O + O_2 \qquad \textbf{(2)}$$

$$5HIO \rightleftharpoons 2I_2 + 2H_2O + HIO_3 \qquad \textbf{(3)}$$

The HIO intermediate decomposes to iodine and yields oxygen in the presence of light or metal catalyst, *via.* $2HIO \leftrightarrow 2H^+ + 2I^- + O_2$. The formation of I^- further catalyze the decomposition of HIO *via.* $2HIO + 2I^- + 2H^+ \leftrightarrow 2I_2 + 2H_2O$, as summarized in **Eqn. 2.** [11] Whereas it decomposes into iodate and iodine by self-disproportionation in acidic environment without light or metal catalyst, *via.* $2OI^- \leftrightarrow IO_2^- + I^-$, $OI^- + IO_2^- \leftrightarrow IO_3^- + I$-. as summarized in **Eqn. 3**. [6,9]

Supplementary Table 1. The details of simulated systems.

Systems	N_{water}	N_{CH3CN}	$N_{Zn}{}^{2+}$	$N_{Li}{}^{+}$	N_{Cl-}	Box size (/Å^3)
19-5-8	700	85	240	63	543	44.05
19-19-8	700	85	240	240	720	46.1
30-19-8	700	85	378	240	996	50.55
30-0-0	700	0	378	0	756	49.4

N represents number of particles; the unit of the box is Å.

Supplementary Table 2. Concentrations of $ZnCl_2$ and LiCl solutions in different systems.

Systems	m_{ZnCl2}	m_{LiCl}
19-5-8	14.91	3.91
19-19-8	14.91	14.91
30-19-8	23.48	14.91
30-0-0	29.97	0

The concentration is in mol kg^{-1}.

Supplementary Note 2

In the simulation system, the water molecule model uses the SPC/E water model package[12]. Firstly, 5ns NPT ensemble was performed in each simulation system for equilibrium to obtain proper box size and water density at a temperature of 298.15K and a pressure of 1atm. Then 10ns NVT simulation was performed, and the last 5 ns was collected for data analysis. Pressure and temperature were regulated using Nosé-Hoover barometer and Nosé - Hoover thermostat[13], respectively. The Lennard-Jones interactions between particles using the Lorentz-Berthelot[13] combination rule. The short range van der Waals interactions were cut off at 12Å. The electrostatic interactions were computed with particle mesh Ewald method[14]. Periodic boundary conditions were imposed in all directions. Force field parameters of all the species are summarized in Supplementary Table 3.

Supplementary Table 3. Force field parameters for ions, water and acetonitrile.

Atom	ε(kcal·mol^{-1})	σ(Å)	q(au·e^{-1})	References
Zn	0.0325	2.0471	2.0000	[15]
Li	0.0183	2.126	1.0000	[16]
Cl	0.1	4.4	−1.0000	[17]
OW	0.1554	3.1655	−0.8476	[18]
H	0.0000	0.0000	0.4238	[18]
Me	0.379	3.6	0.269	[19]
C	0.099	3.4	0.129	[19]
N	0.099	3.3	-0.398	[19]

r_{MeC}=1.46Å, r_{MeN}=2.63Å.

Supplementary Figures:

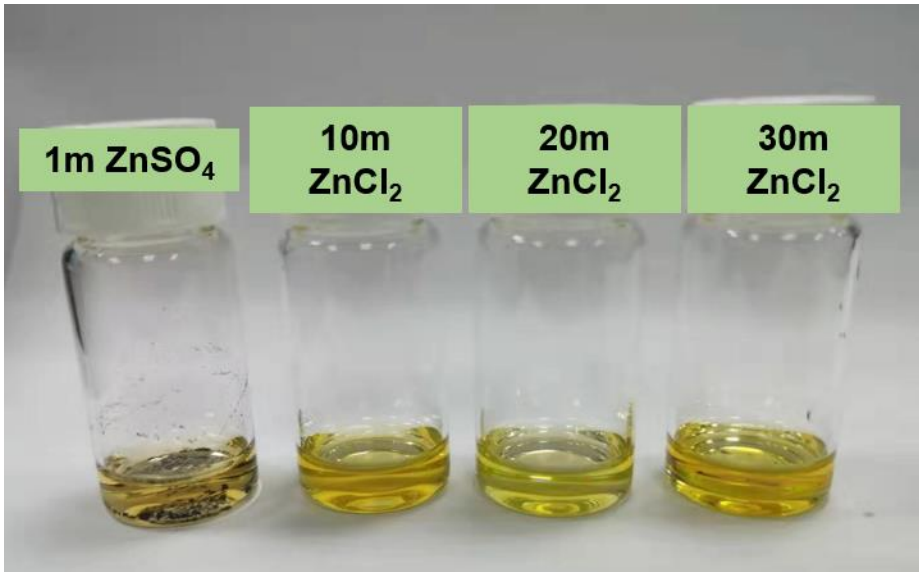

Supplementary Figure 1 | 0.08m ICl in different electrolyte solutions after 5 min retention.

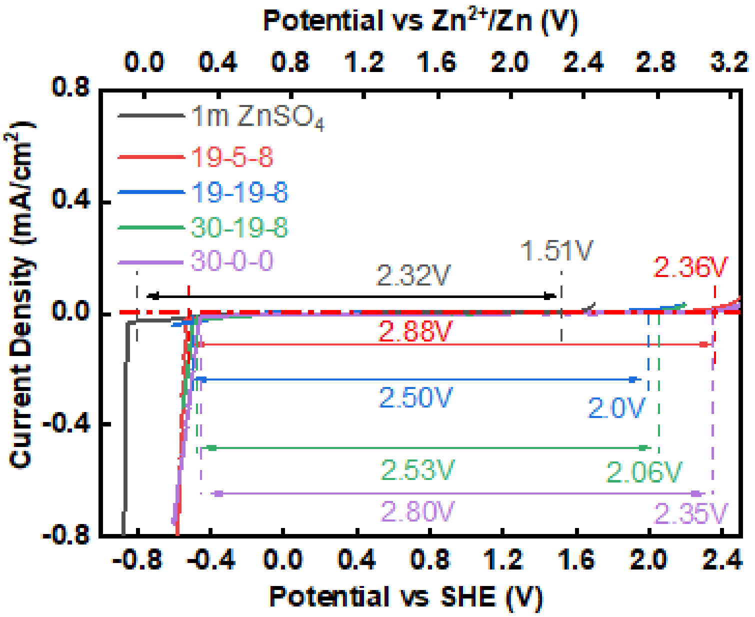

Supplementary Figure 2 | The electrochemical windows of various electrolytes.

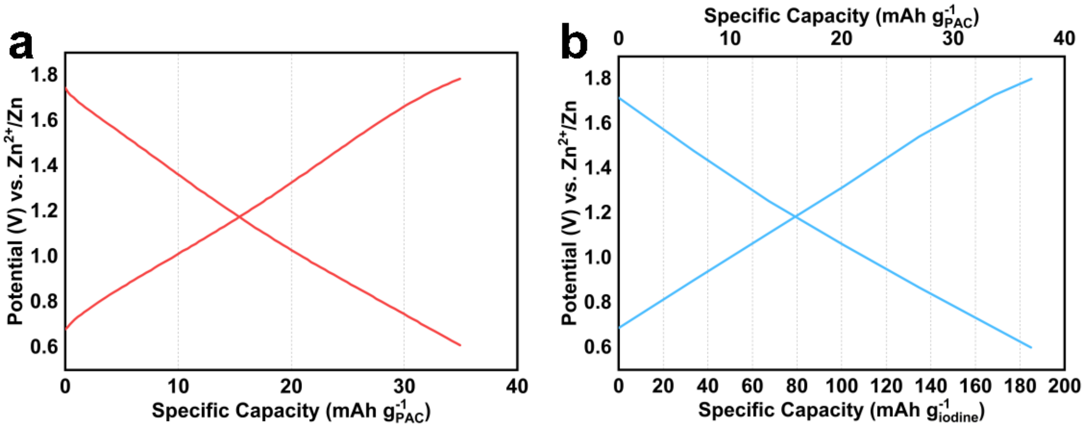

Supplementary Figure 3 | Discharge and charge curves of pure PAC electrode in the Zn||PAC cell. There was no iodine in the electrodes. (a) in the 1 M $ZnSO_4$ electrolyte, and (b) in the 19-19-8 electrolyte. The capacities were provided by the capacitance behavior of the PAC carbon. The specific capacity is about 37 mAh/g based on the PAC mass, corresponding to a capacity of 183 mAh/g based on the average iodine mass of the iodine electrode.

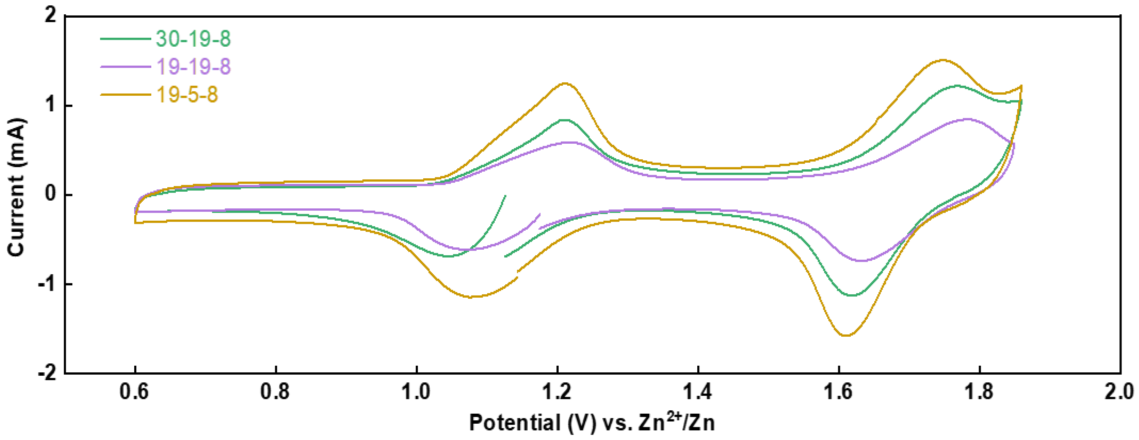

Supplementary Figure 4 | CV curves of 19-5-8, 19-19-8 and 30-19-8 at 0.2mV s^{-1}.

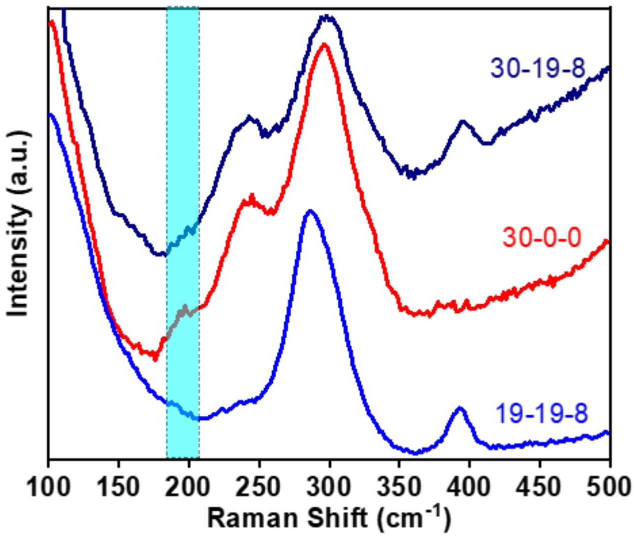

Supplementary Figure 5 | Raman spectroscopy of the electrolytes with 0.5m KI. Signal around 200 cm^{-1} is attributed to Asymmetric Zn-I stretching of $[ZnI_x(OH2)_{4-x}]^{2-x}$.

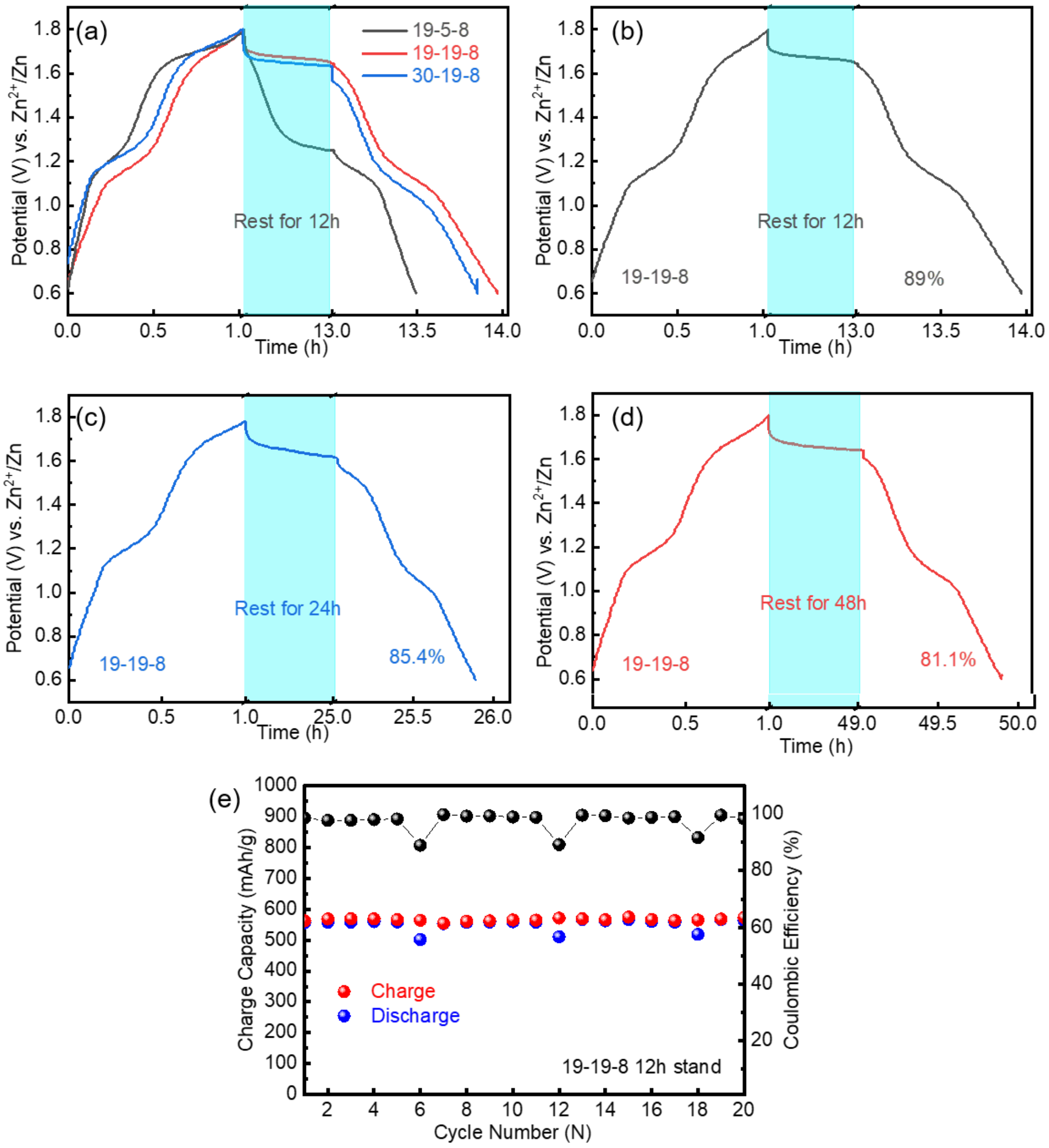

Supplementary Figure 6 | a, Voltage profiles with 12h rest between charge and discharge showing 43%, 89% and 89.3% capacity retention for 19-5-8, 19-19-8 and 30-19-8, respectively and **b-d,** voltage profiles with 12h, 24h, 48h rest between charge and discharge in 19-19-8 systems. **e,** Cycling performance of the Zn-I_2 battery in 19-19-8 electrolyte with 12 hours of intervals for every 5 cycles. Cells were tested at 400 mA g^{-1}.

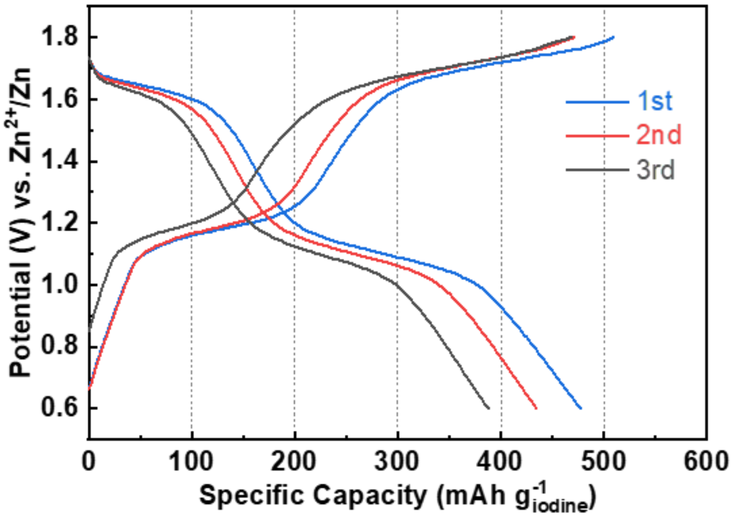

Supplementary Figure 7 | Voltage profiles of 0.1m ZnI_2 + 30m $ZnCl_2$ system. PAC as the cathode only.

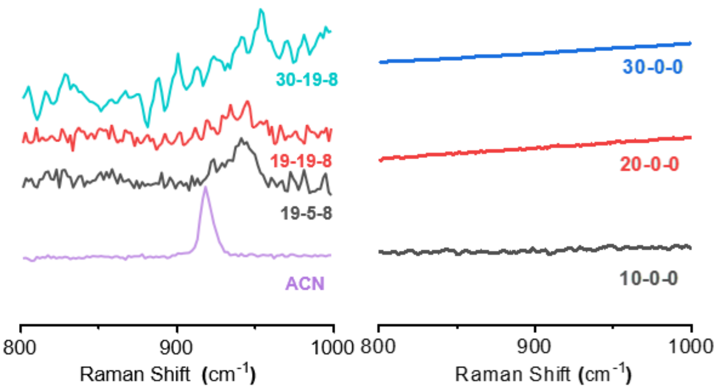

Supplementary Figure 8 | The Raman spectrum of different systems range from 800 cm^{-1} to 1000 cm^{-1}.

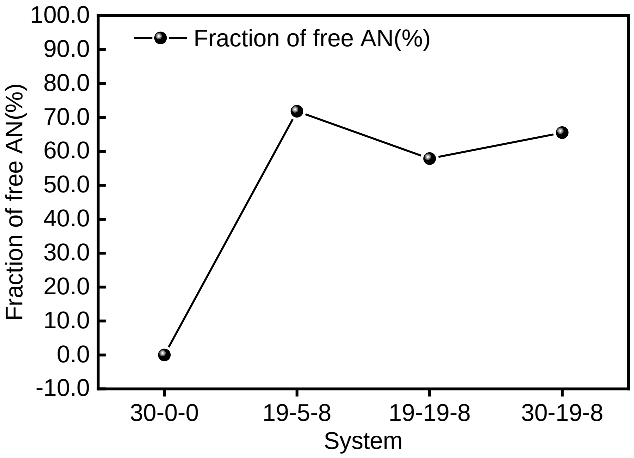

Supplementary Figure 9 | Summary of free AN content extracted from the MD simulation

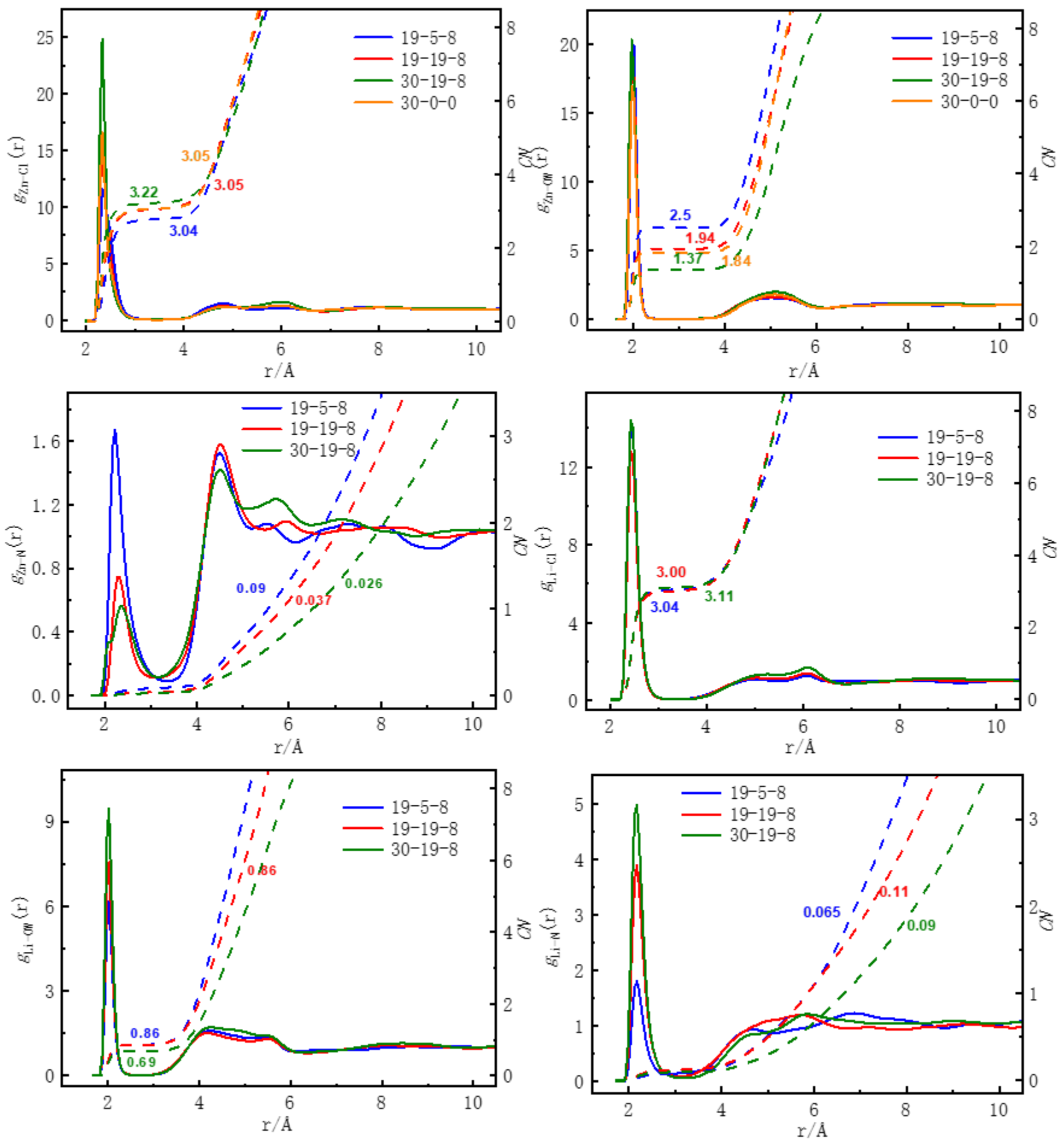

Supplementary Figure 10 | Radical distribution function(RDF) of four systems. The solid lines are the radial correlation function. The dotted lines are the coordination numbers. The Li-Cl, Li-H_2O coordination and the coordination between cation/anion and acetonitrile are much weaker than the Zn-Cl and Zn-H_2O coordination. Note acetonitrile molecules are hardly found in the first solvation sphere, indicating its weak solvation property compares to H_2O.

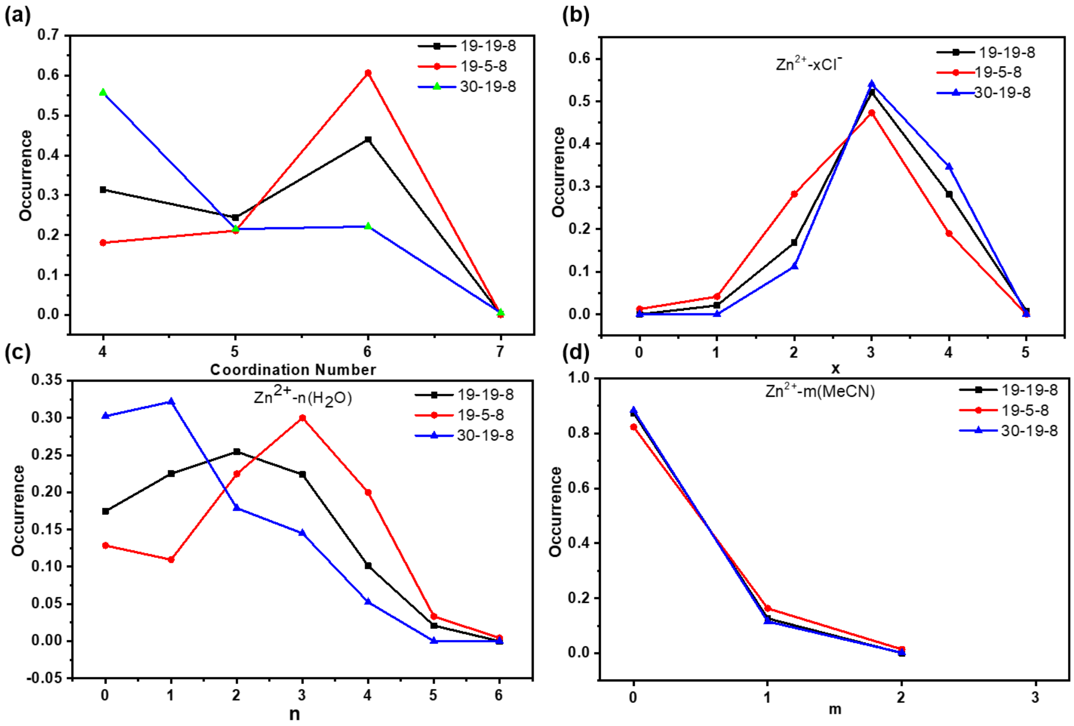

Supplementary Figure 11 | Coordination analysis of Zn^{2+} in different systems. **a**, Total coordination number of Zn^{2+}. **b**, Zn^{2+}-Cl^- coordination. **c**, Zn^{2+}-H_2O coordination. **d**, Zn^{2+}-ACN coordination.

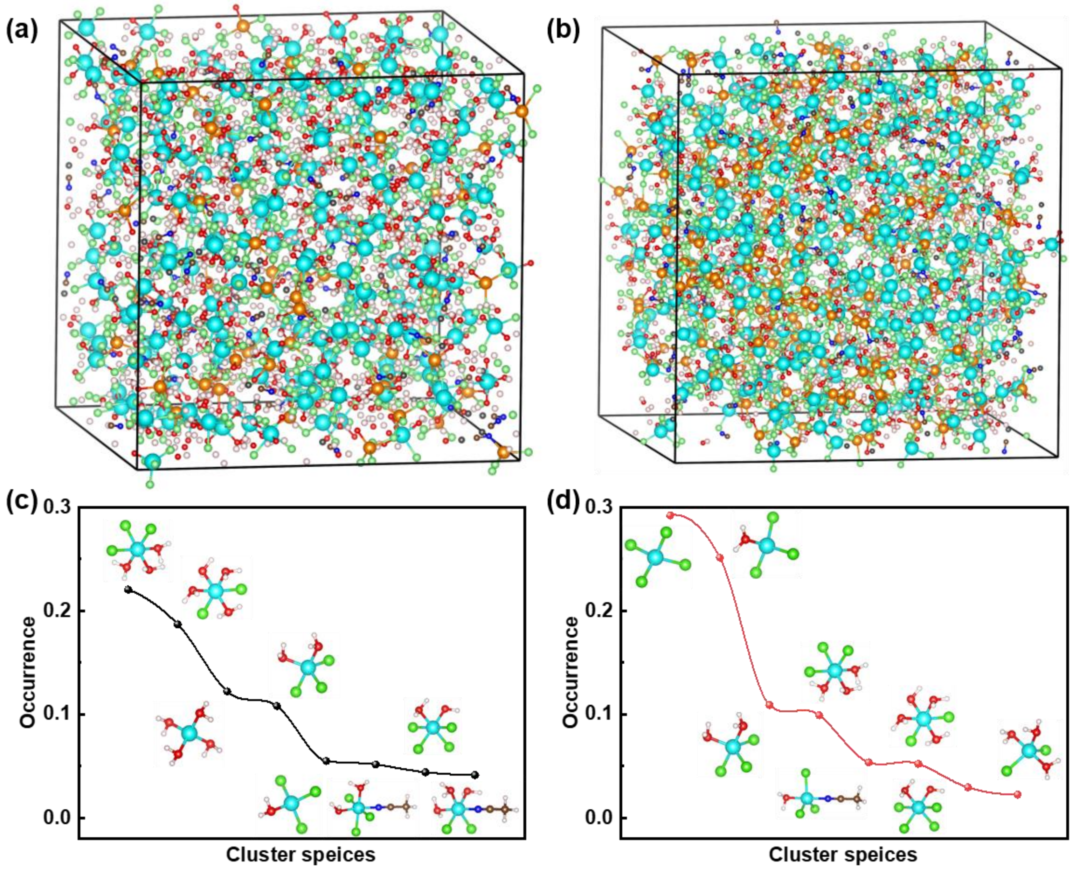

Supplementary Figure 12 | Snapshot of the MD simulation box and their clusters distribution for 19-5-8(**a, c**) and 30-19-8(**b, d**) systems. Cyan-blue atom is Zn^{2+}, green atom is Cl^{-}, yellow atom is Li^{+}, red atom is O, dark blue atom is N, brown atom is C, gray atom is Me, and white atom is H.

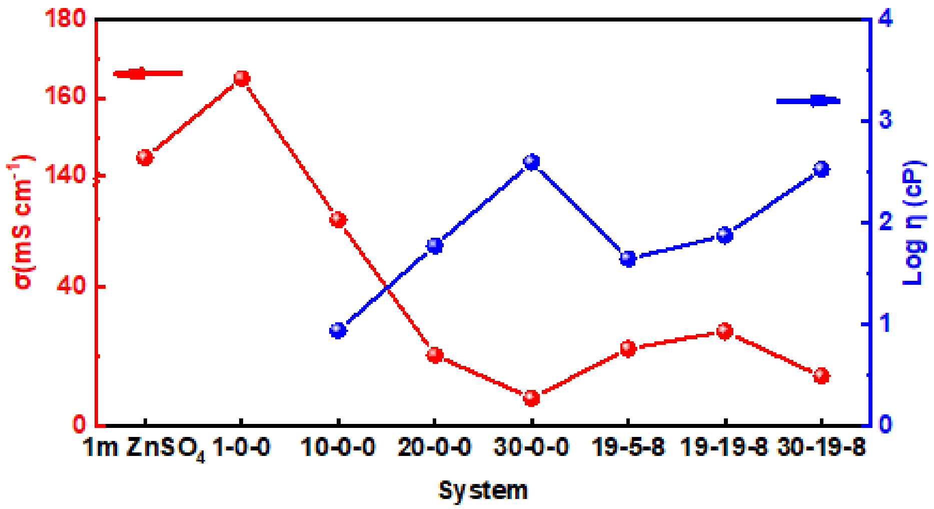

Supplementary Figure 13 | The concentration dependence of viscosity and ionic conductivity at 25 °C for pure $ZnCl_2$ solutions and the bisalt electrolytes.

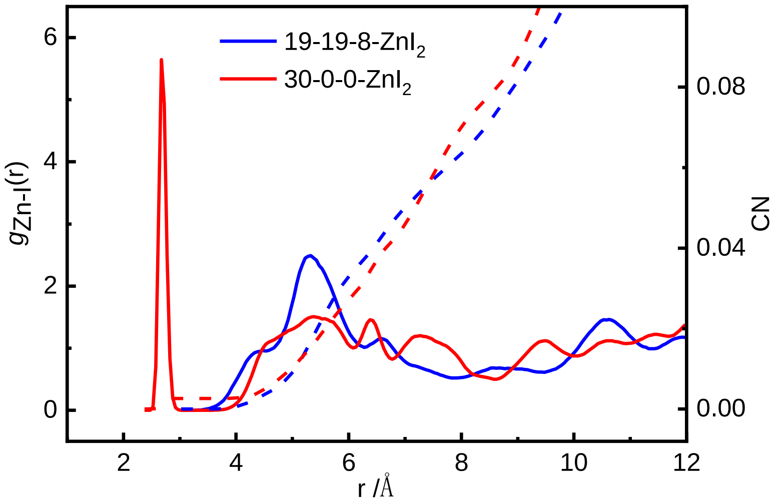

Supplementary Figure 14 | Radical distribution function(RDF) of ZnI_2 in different systems. The solid lines are the radial correlation function. The dotted lines are the coordination numbers.

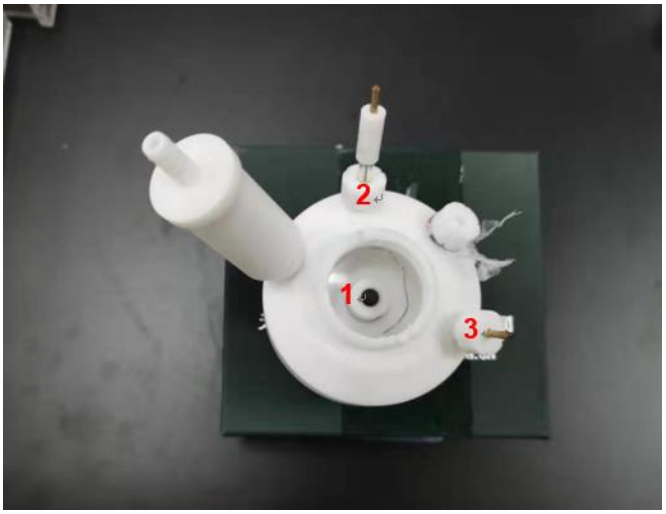

Supplementary Figure 15 | The structure of in-situ Raman electrolytic device. 1# is the location of the working electrode, 2# is the Ag/AgCl reference electrode and 3# is the Pt counter electrode, respectively.

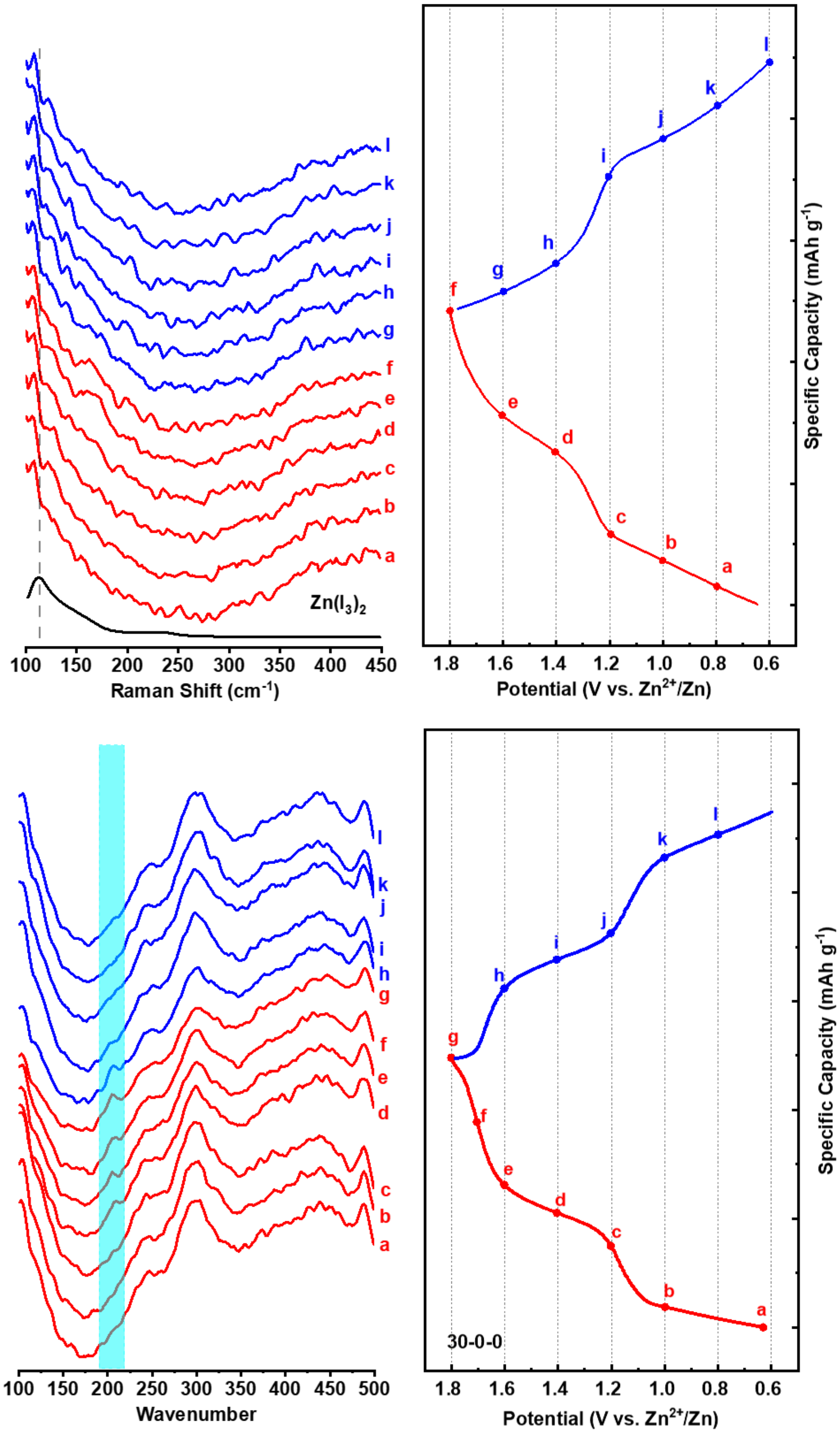

Supplementary Figure 16 | In-situ Raman spectroscopy in 1m $ZnSO_4$ electrolyte(up) and in 30-0-0 system(down) and their charge-discharge curves.

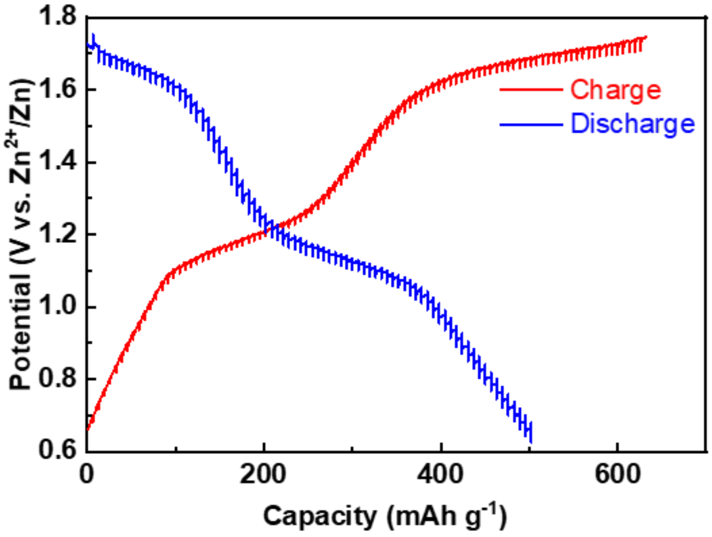

Supplementary Figure 17 | Charge-discharge GITT curves of 19-19-8.

The diffusion coefficient D in the active material can be estimated according to the following equation:

$$D = (4/\pi\tau)\,(n_m V_m/S)^2(\Delta E_s/\Delta E_t)^2 \qquad \textbf{(4)}$$

Where, τ is the constant current pulse duration; n_m and V_m are the moles and molar volume of PAC, respectively; S is the electrode-electrolyte interface area; ΔE_s and ΔE_t are the change in the steady state voltage and overall cell voltage after the application of a current pulse in a single step GITT experiment, respectively[20].

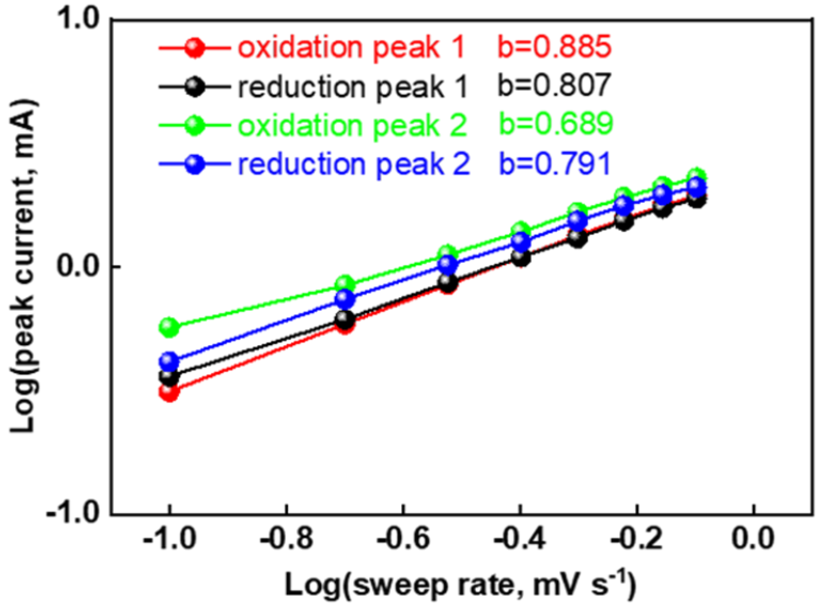

Supplementary Figure 18 | The plots of log *i* versus log *v*, data was collected from CV curves. The b-value are calculated from the slope of the plots.

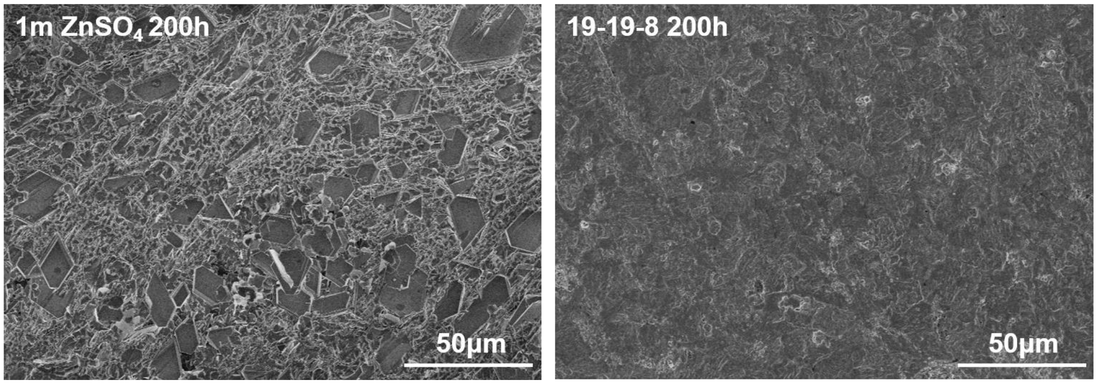

Supplementary Figure 19 | SEM images of the Zn electrodes cycled for 200h (100 cycles) at 1 mA cm^{-2} in 1 m $ZnSO_4$ and 19-19-8, respectively.

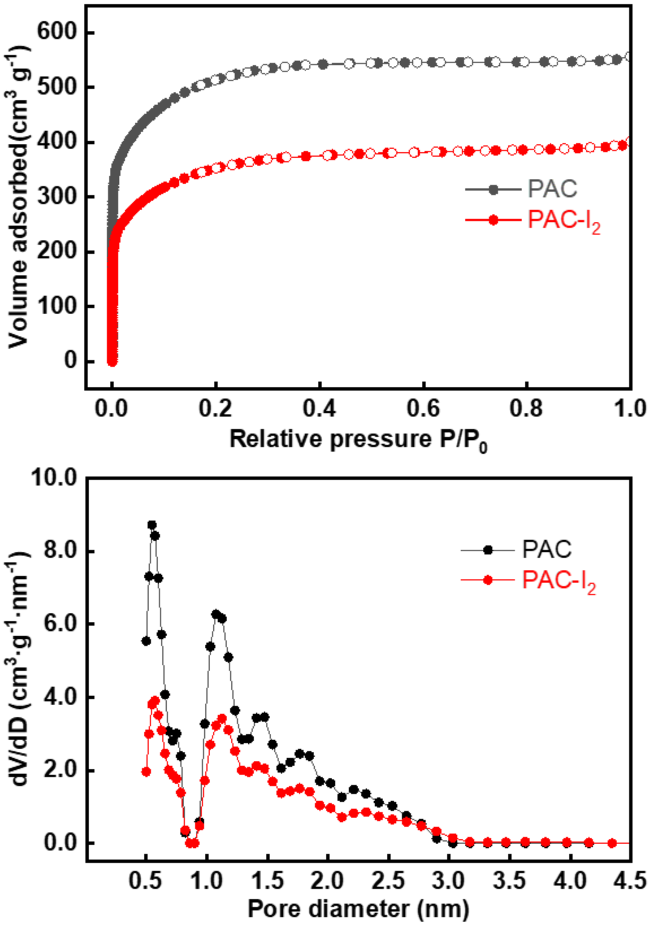

Supplementary Figure 20 | Brunauer–Emmett–Teller surface area calculated from nitrogen adsorption–desorption isotherms for PAC electrodes with/without iodine. The surface area reduced from 2072 to 1408 cm^2 g^{-1} after iodine loaded.

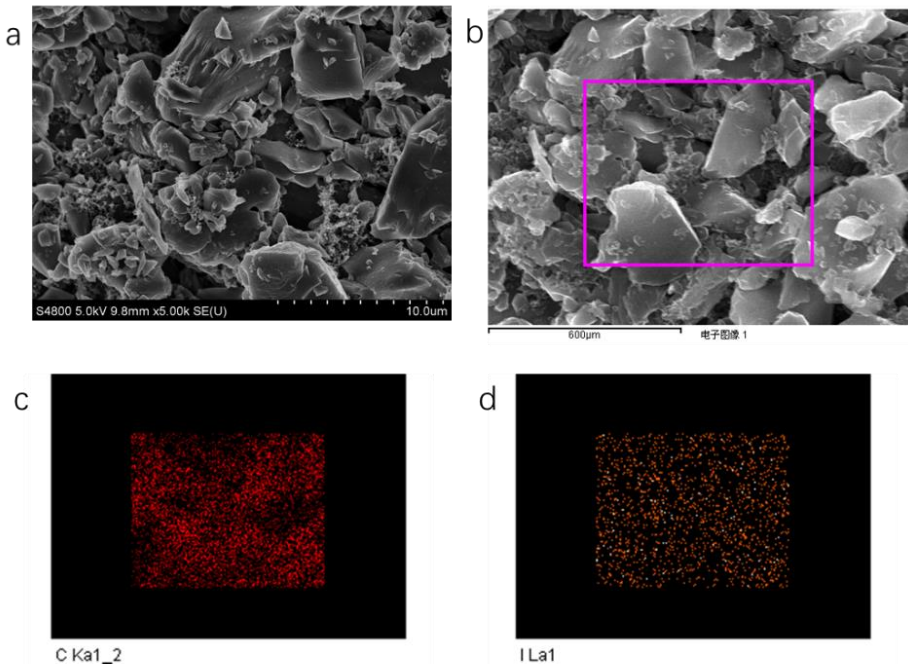

Supplementary Figure 21 | (a) SEM image of the iodine electrode. (b-d) EDS mapping of the iodine electrode to show the homogenously distributed carbon and Iodine.

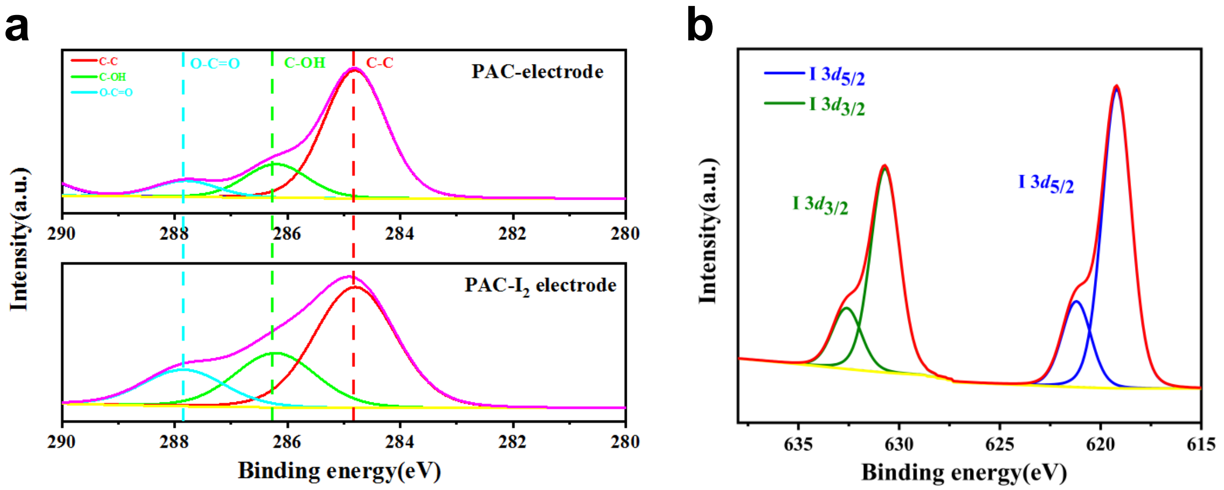

Supplementary Figure 22 | a, C 1s region of XPS spectra of PAC and PAC-I2 composite. **b,** I 3*d* core level spectrum of PAC-I2 composite.

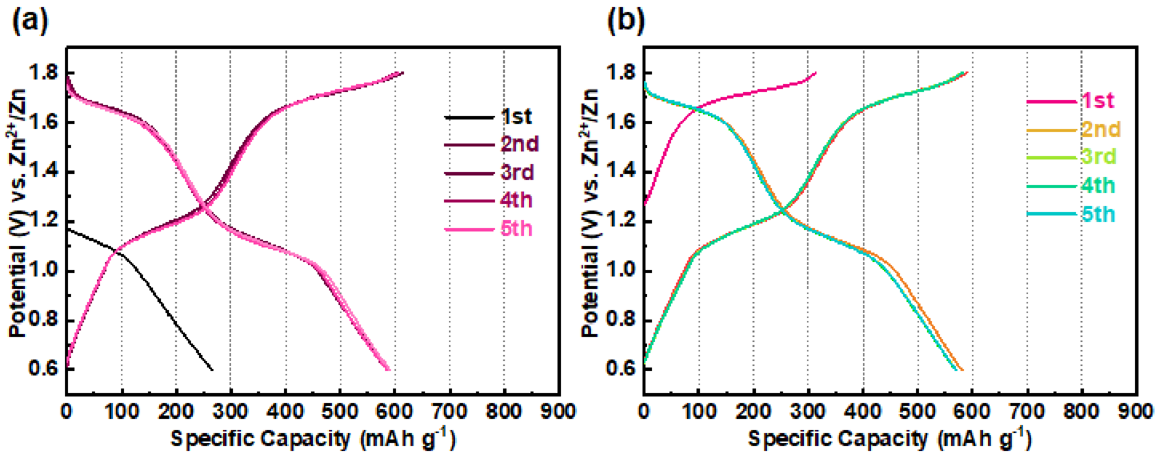

Supplementary Figure 23 | The galvanostatic charge/discharge profiles of 19-19-8 activated by (a) discharge first, and (b) charge first.

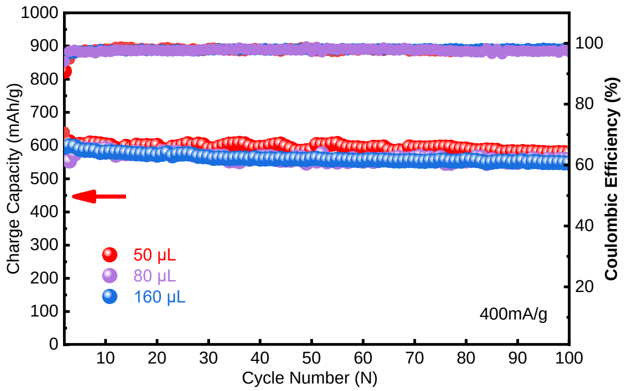

Supplementary Figure 24 | Cycle performance of 19-19-8 system with different electrolyte volume.

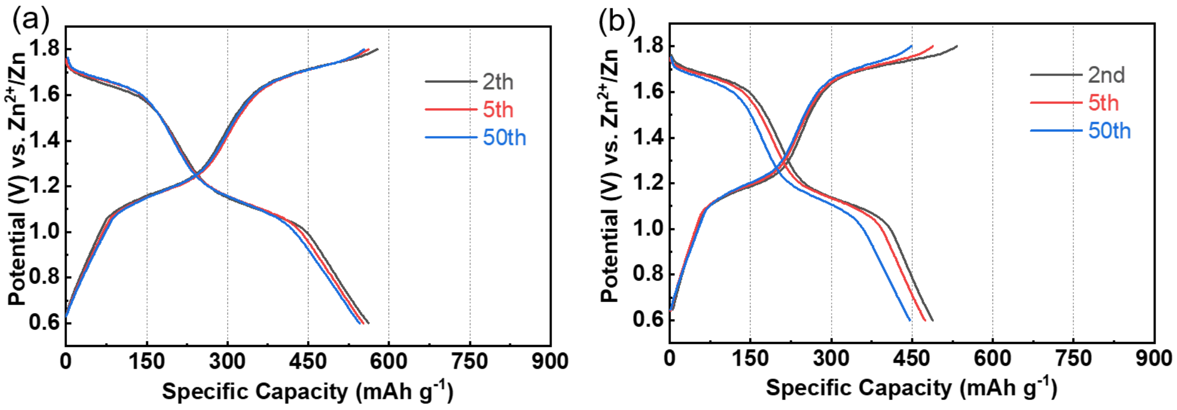

Supplementary Figure 25 | Voltage profiles of the Zn-I_2 battery with the I_2/N-doped carbon electrode and the 19-19-8 electrolyte. (a) iodine areal loading of 7 mg cm^{-2}. (b) iodine areal loading of 9.5 mg cm^{-2}. The N-doped carbon was prepared by carbonization of MOF (ZIF8) at 1000 °C. The iodine ratio in the I_2/N-doped carbon is 45 wt%.

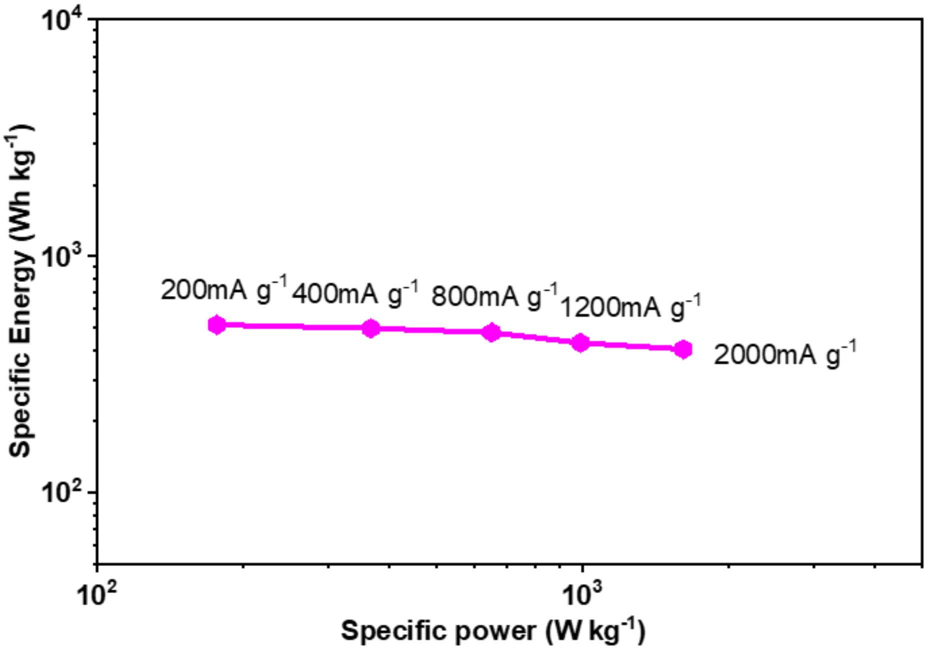

Supplementary Figure 26 | Ragone plot of the four-electron Zn-I_2 battery. The specific energy is based on the total mass of iodine and zinc consumed on anode.

Supplementary References

1 Kolthoff, I.M., and Jordan, J. (1953). Amperometric Titration and Voltammetric Determination of Iodide. Anal. Chem. *25,* 1833–1837.

2 Kolthoff, I. M. & Jordan, J. Amperometric Titration and Voltammetric Determination of Iodide. *Anal. Chem.* **25**, 1833–1837 (1953).

3 Anson, F. C. & Lingane, J. J. Anodic Chronopotentiometry with Platinum and Gold Electrodes. The Iodide-Iodine-Iodate System. *J. Am. Chem. Soc*. **79**, 1015–1020 (1957).

4 Beran, Premysl. & Bruckenstein, Stanley. Voltammetry of iodine(I) chloride, iodine, and iodate at rotated platinum disk and ring-disk electrodes. *Analytical Chemistry* **40**, 1044–1051 (1968).

5 Fialkov, Ya. A. Interhalogen compounds as complex-formers. *Russ Chem Bull* **3**, 847–855 (1954).

6. Philbrick, F. A. The Hydrolysis of Iodine Monochloride. *J. Am. Chem. Soc*. **56**, 1257–1259 (1934).

7 Whitaker, R. D., Ambrose, J. R. & Hickam, C. W. Iodine monochloride and iodine trichloride complexes with heterocyclic amines. *Journal of Inorganic and Nuclear Chemistry* **17**, 254–256 (1961).

8 Wang, Y. L., Nagy, J. C. & Margerum, D. W. Kinetics of hydrolysis of iodine monochloride measured by the pulsed-accelerated-flow method. *J. Am. Chem. Soc.* **111**, 7838–7844 (1989).

9 Bichsel, Y. & von Gunten, U. Oxidation of Iodide and Hypoiodous Acid in the Disinfection of Natural Waters. *Environ. Sci. Technol*. **33**, 4040–4045 (1999).

10 Urbansky. E. T., Cooper B. T. & Margerum D. W., Disproportionation Kinetics of Hypoiodous Acid As Catalyzed and Suppressed by Acetic Acid−Acetate Buffer. *Inorg. Chem.* **36**, 1338-1344 (1997)

11 Downs, A. J. & Adams, C. J. The Chemistry of Chlorine, Bromine, Iodine and Astatine: Pergamon Texts in Inorganic Chemistry. *vol. 7* (Elsevier, 2017).

12. Todorov, I. T., Smith, W., The DL_POLY_4 User Manual, Version 4.06, STFC Daresbury laboratory, Daresbury, Warrington WA4 4AD Cheshire, UK (2014).

13. Hoover, W. G. Canonical dynamics: Equilibrium phase-space distributions. *Phys. Rev. A* **31**, 1695–1697 (1985).

14. Essmann, U. et al. A smooth particle mesh Ewald method. *The Journal of Chemical Physics* **103**, 8577–8593 (1995).

15. Babu, C. S. & Lim, C. Empirical Force Fields for Biologically Active Divalent Metal Cations in Water. *J. Phys. Chem. A* **110**, 691–699 (2006).

16. Åqvist, J. Ion-water interaction potentials derived from free energy perturbation simulations. *J. Phys. Chem.* **94**, 8021–8024 (1990).

17. Smith, D. E. & Dang, L. X. Computer simulations of NaCl association in polarizable water. *The Journal of Chemical Physics* **100**, 3757–3766 (1994).

18. Berendsen, H. J. C., Grigera, J. R. & Straatsma, T. P. The missing term in effective pair potentials. *J. Phys. Chem*. **91**, 6269–6271 (1987).

19. Edwards, D. M. F., Madden, P. A. & McDonald, I. R. A computer simulation study of the dielectric properties of a model of methyl cyanide: I. The rigid dipole case. *Molecular Physics* **51**, 1141–1161 (1984).

20. Kundu, D., Adams, B. D., Duffort, V., Vajargah, S. H. & Nazar, L. F. A high-capacity and long-life aqueous rechargeable zinc battery using a metal oxide intercalation cathode. *Nat Energy* **1**, 1–8 (2016).
